# The Endogenous Th17 Response in NO_2_-Promoted Allergic Airway Disease Is Dispensable for Airway Hyperresponsiveness and Distinct from Th17 Adoptive Transfer

**DOI:** 10.1371/journal.pone.0074730

**Published:** 2013-09-19

**Authors:** Rebecca A. Martin, Jennifer L. Ather, Rebecca Daggett, Laura Hoyt, John F. Alcorn, Benjamin T. Suratt, Daniel J. Weiss, Lennart K. A. Lundblad, Matthew E. Poynter

**Affiliations:** 1 Vermont Lung Center, Division of Pulmonary Disease and Critical Care, University of Vermont, Burlington, Vermont, United States of America; 2 Division of Pulmonology, Department of Pediatrics, Children’s Hospital of Pittsburgh of UPMC, Pittsburgh, Pennsylvania, United States of America; National Jewish Health, United States of America

## Abstract

Severe, glucocorticoid-resistant asthma comprises 5-7% of patients with asthma. IL-17 is a biomarker of severe asthma, and the adoptive transfer of Th17 cells in mice is sufficient to induce glucocorticoid-resistant allergic airway disease. Nitrogen dioxide (NO_2_) is an environmental toxin that correlates with asthma severity, exacerbation, and risk of adverse outcomes. Mice that are allergically sensitized to the antigen ovalbumin by exposure to NO_2_ exhibit a mixed Th2/Th17 adaptive immune response and eosinophil and neutrophil recruitment to the airway following antigen challenge, a phenotype reminiscent of severe clinical asthma. Because IL-1 receptor (IL-1R) signaling is critical in the generation of the Th17 response *in vivo*, we hypothesized that the IL-1R/Th17 axis contributes to pulmonary inflammation and airway hyperresponsiveness (AHR) in NO_2_-promoted allergic airway disease and manifests in glucocorticoid-resistant cytokine production. IL-17A neutralization at the time of antigen challenge or genetic deficiency in IL-1R resulted in decreased neutrophil recruitment to the airway following antigen challenge but did not protect against the development of AHR. Instead, IL-1R-/- mice developed exacerbated AHR compared to WT mice. Lung cells from NO_2_-allergically inflamed mice that were treated *in vitro* with dexamethasone (Dex) during antigen restimulation exhibited reduced Th17 cytokine production, whereas Th17 cytokine production by lung cells from recipient mice of *in vitro* Th17-polarized OTII T-cells was resistant to Dex. These results demonstrate that the IL-1R/Th17 axis does not contribute to AHR development in NO_2_-promoted allergic airway disease, that Th17 adoptive transfer does not necessarily reflect an endogenously-generated Th17 response, and that functions of Th17 responses are contingent on the experimental conditions in which they are generated.

## Introduction

As of 2010, the Centers for Disease Control and Prevention reported US asthma prevalence at 1 in 12 adults, and 1 in 11 children, the highest ever documented [1]. Within the asthmatic population, 5-7% has severe disease, which is by definition resistant to treatment with glucocorticoids (GC) [2,3]. Despite the relative infrequency of severe asthma, this population represents 40-50% of asthma health care costs [2]. Furthermore, severe asthmatics are a heterogeneous population, which likely reflects diverse underlying pathophysiologic mechanisms [4]. This patient population could benefit from more comprehensive clinical categorization, including the characterization of functionally relevant biomarkers.

Nitrogen dioxide (NO_2_) is a toxic byproduct of combustion, a component of air pollution, and an endogenously-generated mediator of inflammation [5]. Exposure to NO_2_ correlates with asthma severity, disease exacerbation, risk of adverse outcomes in asthma, and development of asthma in adolescence [6-8]. NO_2_ exposure is capable of sensitizing mice to the innocuous antigen ovalbumin (OVA) [9]. This model reflects the impact of short-term exposure to moderate concentrations of NO_2_ in the development of inhalational allergy, taking into account the increased resistance to NO_2_-mediated inflammation of mice compared to humans [5,10]. Following challenge with OVA antigen, mice exposed to NO_2_ and OVA during sensitization induce pulmonary inflammation including neutrophil and eosinophil recruitment to the airway, the generation of a mixed Th2/Th17 adaptive immune response, and the development of airway hyperresponsiveness (AHR), arguably the most relevant endpoint clinically [9,11,12]. This inflammatory profile is reminiscent of that observed in severe asthma [13].

Traditionally, asthma has been considered a Th2-mediated disease, but other immunologic mechanisms likely contribute, notably the Th17 adaptive immune response. Increased IL-17 can be measured from the BAL and sputum of asthmatic subjects compared with healthy controls [14,15]. Furthermore, IL-17 levels and increased CD4^+^ Th17 cells in the peripheral blood correlate with asthma severity [16,17]. Th17 cells are a distinct subset of CD4^+^ T-cells capable of producing IL-17A, IL-17F, IL-22, and GM-CSF, which are regulated in part by the Th17-specific transcription factor RORγt [18]. We and others have demonstrated that IL-1R signaling is critical in the development of Th17 responses [19-23], including that generated in NO_2_-promoted allergic airway disease [11]. While additional cell types can produce IL-17 [24], the CD4^+^TCRβ^+^ population of cells was recently identified as the relevant IL-17A-producing cell population in NO_2_-promoted allergic airway disease following antigen challenge [11].

While evidence exists indicating that IL-17A may downregulate inflammation [25,26], IL-17A is thought to promote asthma pathogenesis by stimulating the production of inflammatory cytokines – including CXC chemokines capable of recruiting neutrophils, inducing smooth muscle contraction, increasing mucus production, and promoting GC resistance [15,27-31]. The adoptive transfer of *in vitro*-polarized Th17 cells results in neutrophilic, GC-resistant allergic airway disease following antigen challenge, whereas pulmonary inflammation and AHR following the adoptive transfer of Th2-polarized cells is GC-sensitive [32]. Neutrophils contribute to asthma pathogenesis by releasing enzymes that potentiate tissue damage, oxidant formation, and mucus secretion [33,34]. The recruitment of neutrophils is associated with more severe asthma, including frequency of symptoms and reduced pulmonary function [14].

In contrast to the Th17 response, the Th2 immune response is characterized by eosinophil recruitment to the airway, mucus production, increased expression of the mucin-associated genes *Gob5* and *Muc5ac*, and the production of Th2 and Th2-promoting cytokines IL-4, IL-5, IL-13, and IL-25 [13,35,36]. IL-4 signaling at the time of sensitization is critical for generating the Th2 response [37,38]. Signaling through IL-4Rα pathway results in STAT6 phosphorylation and upregulation of the Th2-specific transcription factor, *Gata3* [39,40]. Because IL-4Rα is a component of both the IL-4 and IL-13 receptors, mice that are genetically deficient in STAT6 are generally unable to respond to IL-4 and IL-13 [39].

The process of NO_2_-promoted allergic sensitization likely involves downstream generation of endogenous danger-associated molecular patterns (DAMPs) by airway epithelial cells, activating antigen presenting cells (APCs) to produce inflammatory mediators including activated IL-1β that results in the development of a Th17 response [5,9,11,12,41,42]. Despite our insight into the mechanisms required for generating the Th17 response in NO_2_-promoted allergic airway disease, the functional relevance of the Th17 response remains unclear. Because the Th17 response is necessary and sufficient to drive pulmonary inflammation and AHR in multiple animal models of asthma [13,32,43], we hypothesized that the IL-1R-dependent Th17 response in NO_2_-promoted allergic asthma would contribute to pulmonary inflammation and AHR development following antigen challenge. Given the previously observed resistance of the Th17 response to inhibition by the GC dexamethasone (Dex) [32], we further hypothesized that the endogenously-generated IL-1R-dependent Th17 response in NO_2_-promoted allergic airway disease would exhibit Dex resistance. Whereas IL-17A neutralization at challenge and genetic deficiency in IL-1R resulted in the inhibition of neutrophil recruitment to the airway following antigen challenge, AHR was either not affected or exacerbated. These results are surprising, and given Th17’s pathologic role in GC resistance, we sought to compare the endogenously-generated Th17 response to that manifest following the adoptive transfer of *in vitro*-polarized Th17 OTII T-cells. We found that the endogenously generated Th17 response in NO_2_-promoted allergic airway disease was both quantitatively and qualitatively different from that generated following Th17 adoptive transfer, exhibiting sensitivity to both Dex and IL-1R inhibition with recombinant IL-1 receptor antagonist (anakinra) during *ex vivo* lung restimulation in the presence of antigen. In contrast, the Th17 response generated by lung cells from recipients of the Th17 adoptive transfer was resistant to Dex and anakinra. These data suggest that the adoptive transfer model for studying Th17 responses in allergic airway disease is not necessarily representative of the endogenously-generated Th17 response and that the Th17 response does not represent a single entity, but a response with functional variability that is contingent on the experimental conditions in which it is generated.

## Materials and Methods

### Mice

Female C57BL/6, IL-1R-/-, STAT6-/- (Jackson Laboratories, Bar Harbor, ME), and female OTII transgenic mice (C57BL/6 background; bred at the University of Vermont) were 6-8 weeks old at the beginning of experimentation. Studies were approved by the Institutional Animal Care and Use Committee of the University of Vermont under the permits 09-050, 12-018, and 12-020. Mice were housed in an AAALAC approved facility, maintained on a 12-hour light/dark cycle, and were provided food and water ad libitum. All mice were euthanized with sodium pentobarbitol (200-300 μL by i.p. injection; Wilcox Pharmacy, Rutland, VT) or for pulmonary function assessment, anesthetized with 90 mg/kg sodium pentobarbital. All experiments were conducted with conscious effort to minimize animal suffering.

### NO_2_-promoted allergic sensitization and challenge

For NO_2_-promoted allergic sensitization, a single 1-hour exposure to 15ppm of NO_2_ on day 1 was followed by 30 minutes of nebulized 1% OVA, Fraction V (Sigma-Aldrich, St. Louis, MO) in saline, on days 1, 2, and 3 [11]. All mice were OVA-challenged on days 14, 15, and 16, as described [41]. Analyses were performed at 48 hours after the final OVA challenge, on day 18.

### Antibody treatments

For neutralization of IL-17A, 0.5 mg of neutralizing anti-IL-17A antibody (17F3; BioXCell, West Lebanon, NH) or IgG isotype antibody (MOPC21; BioXCell) was administered by i.p. injection in 500 μL saline on day 13. For inhibition of the Th2 response, 1 mg of neutralizing anti-IL-4 antibody (11B11; BioXCell) was administered in a single i.p. injection delivered on day 0, and some mice received an additional 1 mg of neutralizing anti-IL-4 antibody on day 13. Control mice received 1 mg of IgG control antibody (MOPC21; BioXCell) in a single i.p. injection in 500 μL saline on days 0 and 13.

### *In vitro* polarization and adoptive transfer

Splenocytes and peripheral lymph nodes were harvested from OTII transgenic mice. Single cell suspensions were generated by passing the tissues through a 70 μm nylon mesh filter (BD Biosciences, San Jose, CA) and lymphocytes were enriched by centrifugation through Lymphocyte Separation Medium (MP Biomedicals, Irvine, CA). CD4^+^ T-cells were isolated via magnetic negative selection following manufacturer’s instructions (StemCell Technologies, Vancouver, BC). Following 3 wash steps, cells were plated at 1x10^6^ cells/mL in CD4^+^ complete medium (5% FBS (Cell Generation, Fort Collins, CO), pen/strep, L- glutamine, folic acid, glucose, and 2-ME in RPMI-1640 (Gibco, Grand Island, NY), containing 2 μg/mL anti-CD28; BD Biosciences) in wells pre-coated with 5 μg/mL anti-CD3 (BD Biosciences). Th17 differentiation was performed using 10 μg/mL anti-IFNγ (R&D Systems, Minneapolis, MN), 10 μg/mL anti-IL-4 (R&D Systems), 30 ng/mL IL-6 (R&D Systems), 10 ng/ml IL-23 (R&D Systems), and 1 ng/mL TGFβ (R&D Systems). Th2 T-cells were differentiated in 10 μg/mL anti-IFNγ (R&D Systems), 30 ng/mL IL-4 (R&D Systems), and 20 units/mL IL-2 (R&D Systems). Cell cultures were split on day 3 and resuspended in fresh media containing appropriate polarizing and T-cell stimulating mediators. *In vitro* differentiated cells were harvested on day 6 and supernatants were validated for appropriate cytokine production by ELISA. Washed cells were resuspended at 1x10^7^ cells/ml in PBS and 2x10^6^ cells were adoptively-transferred into the retroorbital sinus of isoflurane-anesthetized female C57BL/6 mice. Recipient mice were antigen-challenged immediately following adoptive transfer and on each of the next two days for 30 minutes with aerosolized OVA. Mice were analyzed 24 hours following the final antigen exposure.

### Assessment of airway hyperresponsiveness

Mice were anaesthetized with i.p. sodium pentobarbital (90mg/kg), the trachea were cannulated and the mice were connected to a flexiVent™ computer controlled small animal ventilator (SCIREQ, QC, Canada), as previously described [44-46]. Mice were ventilated at 200 breaths/minute and paralyzed with an i.p. injection of pancuronium bromide (0.8 mg/kg). The animals were stabilized over about ten minutes of regular ventilation at a positive end-expiratory pressure (PEEP) of 3 cmH _2_O. A standard lung volume history was then established by delivering two total lung capacity maneuvers (TLC) to a pressure limit of 25 cmH _2_O and holding for three seconds. Next, two baseline measurements of respiratory input impedance (*Z*_*rs*_) were obtained. This was followed by an inhalation of aerosolized PBS (control) for 10 seconds, achieved by an in-line piezo electric nebulizer (Aeroneb, Aerogen, Galway, Ireland). *Z*_*rs*_ was then measured every 10 seconds for 3 minutes (18 measurements of *Z*_*rs*_ in total). This complete sequence of maneuvers and measurements was then repeated for aerosol exposures to three ascending doses of aerosolized methacholine. Data were fit to the single-compartment model [47] to provide values for resistance (R), reflecting constriction in the lungs, and elastance (E), reflecting the elastic rigidity of the lungs. R and E are presented as the percentage change from baseline, as measured at the beginning of the protocol.

### BAL collection and processing

Lungs were lavaged with 1 mL DPBS (Sigma-Aldrich, St. Louis, MO). The BAL fluid was centrifuged, and the total cells in the pellet were resuspended in PBS and enumerated by counting with an Advia 120 Hematology System (Bayer HealthCare, Leverkusen, Germany). Differential analysis was performed by cytospin and H&E stain from approximately 200 cells per slide.

### Preparation of lung single cell suspensions

Lung cells were prepared in the lung dissociation kit (Miltenyi Biotec, Auburn, CA) and a GentleMACs dissociator (Miltenyi Biotec), according to manufacturer’s instructions. Four rounds of program m_lung_01 were used before the first incubation, a single round of program “B” was used between incubations, and m_lung_02 was used before lysing red blood cells with ACK buffer (8,024 mg/l NH_4_Cl, 1,001 mg/l KHCO_3_, 7.722 mg/l EDTA·Na_2_ 2H_2_O).

### *In vitro* antigen restimulation and cytokine quantitation

1×10^6^ cells/ml in CD4^+^ complete media were activated with 400 μg/ml (unless otherwise noted) for 96 hours. Where indicated, samples were incubated in the presence of 10^-8^ M dexamethasone (Sigma, St. Louis, MO) and/or 200 ng/mL anakinra (Biovitrum, Stockholm, Sweden). Supernatants were analyzed by ELISA (R&D Systems) or Luminex-based multiplex assay (Millipore, Billerica, MA).

### Quantitative RT-PCR

Total RNA was extracted from snap frozen whole lungs or the single large lung lobe using the PrepEase RNA Isolation kit (USB Corp., Cleveland, OH) and reverse transcribed to cDNA using the iScript kit (Bio-Rad, Hercules, CA). Primers were designed for mouse *Cxcl5* (5’-TCCTCAGTCATAGCCGCAAC-3’ and 5’-TGCGAGTGCATTCCGCTTA-3’, *Lcn2* (5’-CAATGTCACCTCCATCCTGGT-3’ and 5’-ACCATGGCGAACTGGTTGTA-3’, *Muc5ac* (5’-ACCGCTCTGATGTTCCTCAC-3’ and 5’-AGGACCCCAGACTGGCTAAA-3’), and *Gob5* (5’-AAGCCAACCACTCCCATGAC-3’ and 5’-TGCGAAAGCATCAACAACAC-3’) using NCBI Primer-BLAST and synthesized by Integrated DNA Technologies (Coralville, IA). RT-PCR was performed using SYBR Green Supermix (Bio-Rad) and normalized to *Gapdh* (5’-ACGACCCCTTCATTGACCTC-3’ and 5’-TTCACACCCATCACAAACAT-3’) or *Actb* (5’-TCCTTCGTTGCCGGTCCACA-3’ and 5’-CGTCTCCGGAGTCCATCACA-3’) using the ΔΔC_T_ method, as previously described [48].

### Statistical Analysis

Data were analyzed by two-tailed unpaired student’s t-test, one-way ANOVA, or two-way ANOVA using GraphPad Prism 4 for Mac (GraphPad Software, La Jolla, CA). Statistically significant (p < 0.05) results by ANOVA were further analyzed by Bonferroni or Tukey post-hoc test. 

## Results

### IL-17A is required for airway recruitment of neutrophils but not AHR in NO_2_-promoted allergic airway disease

**Figure 1:**
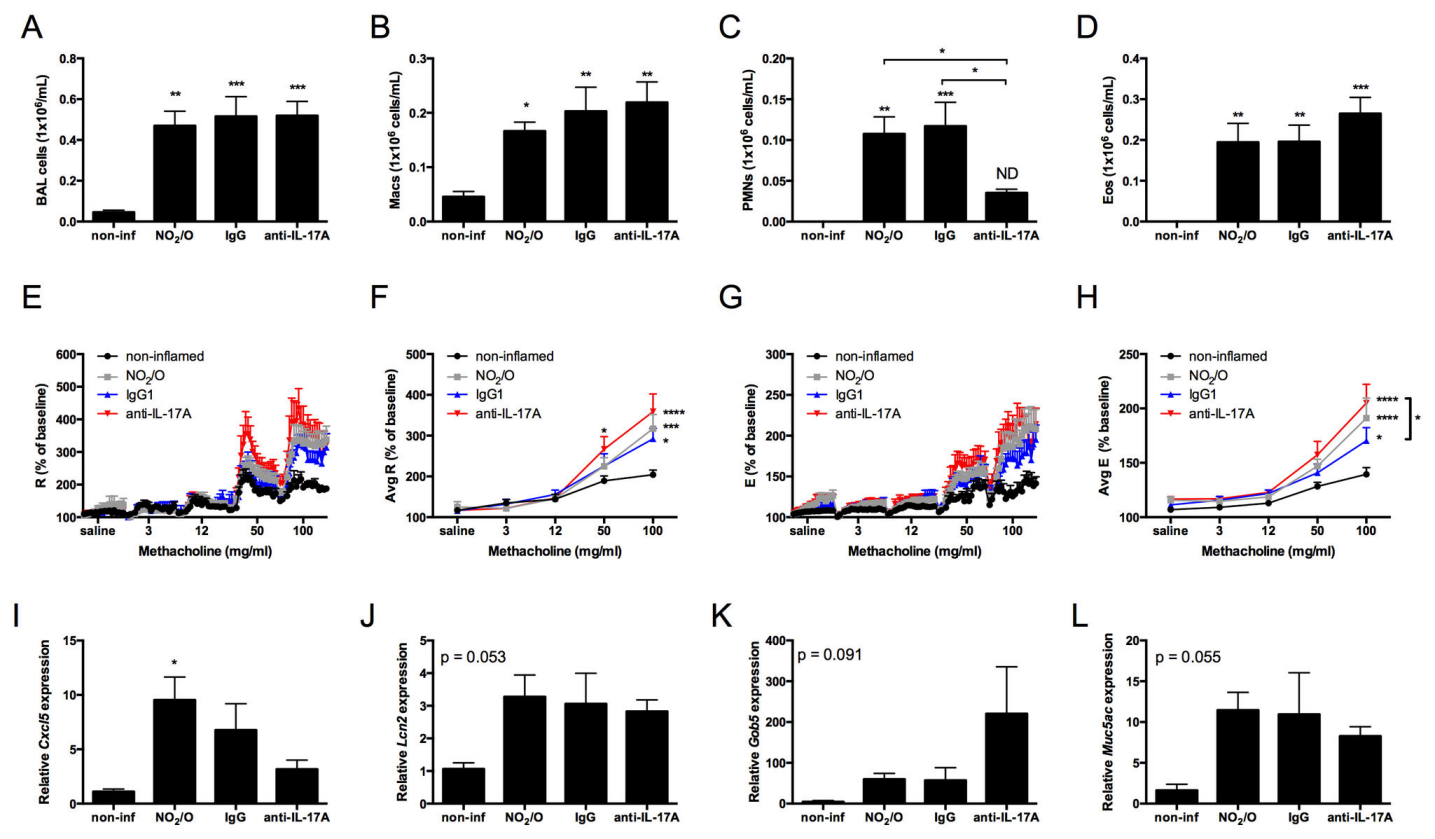
IL-17A is required for airway recruitment of neutrophils but not AHR in NO_2_-promoted allergic airway disease. C57BL/6 mice were exposed to 15ppm NO_2_ for 1 hour on day 1. All mice were exposed to OVA on days 1-3 and again during challenge on days 14-16. Treatment groups received either IgG isotype control antibody or anti-IL-17A neutralization antibody one day prior to antigen challenge on day 13. 48 hours following the final antigen challenge, BAL total cells (A) were quantified and macrophages (Macs; B), neutrophils (PMNs; C), and eosinophils (Eos; D) were calculated based on cell fractions. Pulmonary function assessment was performed by invasive forced oscillation technique. The percent baseline and average percent baseline per dose methacholine were calculated for resistance (R; E-F) and elastance (E; G-H), as determined by the single-compartment model [47]. Lungs were removed and snap frozen prior to RNA analysis for *Cxcl5* (I)*, Lcn2* (J), *Gob5* (K), and *Muc5ac* (L). Statistics were performed by 1-way ANOVA (A-D and I-L) or 2-way ANOVA (E-H) and Tukey post hoc analysis. **** p < 0.0001, *** p < 0.001, ** p < 0.01, * p < 0.05 compared to non-inflamed unless indicated by brackets. ND, not significantly different from non-inflamed. n = 7 per group (A-H) or n = 5-7 per group (I-L).

IL-17A is recognized to promote pulmonary inflammation in allergic airway disease [13,28,31,49]. Having previously identified factors critical in the generation of the Th17 adaptive immune response in NO_2_-promoted allergic airway disease [11,12,41], we sought to further characterize the functional relevance of the Th17 response in this model by first characterizing AHR and airway inflammation. Following antigen challenge, allergically inflamed mice demonstrated increased total BAL cells, comprised of macrophages, neutrophils and eosinophils (Figure 1A-D). In response to challenge with increasing doses of methacholine (MCh), mice that had been allergically sensitized with NO_2_ and antigen challenged developed a statistically significant increase in resistance (Figure 1E-F) and elastance (Figure 1G-H) compared to non-inflamed control mice. Allergically sensitized mice also demonstrated a statistically significant increase in the expression of the neutrophil-recruiting chemokine *Cxcl5* in the lung (Figure 1I). Trends for increased neutrophil and epithelial derived gelatinase *lipocalin-2* (*Lcn2*; Figure 1J), the mucin-associated gene *Gob5* (Figure 1K), and the mucin gene *Muc5ac* (Figure 1L) were also evident in the lungs of allergically inflamed mice [50,51].

To address the functional contribution of IL-17A in NO_2_-promoted allergic airway disease, we administered anti-IL-17A neutralizing antibody or IgG isotype control antibody one day prior to the first OVA challenge. IgG-treated mice that had been allergically sensitized and antigen challenged exhibited similar cellularity in the BAL and AHR as that of untreated NO_2_-sensitzed and challenged mice. Mice that received neutralizing anti-IL-17A antibody demonstrated no differences in total numbers of BAL cells (Figure 1A), macrophages (Figure 1B), or eosinophils (Figure 1D). However, the number of neutrophils in the BAL of mice treated with anti-IL-17A was significantly decreased in comparison to IgG-treated mice (Figure 1C). Surprisingly, mice that were treated with anti-IL-17A neutralizing antibody at the time of antigen challenge developed similar resistance (Figure 1E-F) and elastance (Figure 1G-H) to that of IgG-treated mice. While elastance that developed in anti-IL-17A treated mice was significantly increased over that of IgG isotype control treated mice, elastance in anti-IL-17A treated mice was not increased above that of untreated NO_2_-sensitized and challenged mice. Gene expression analysis revealed a trend for decreased *Cxcl5* (Figure 1I) and a trend for increased *Gob5* expression (Figure 1K) in the lungs of anti-IL-17A treated mice, whereas *Muc5ac* and *Lcn2* did not differ between IgG isotype control and anti-IL-17A treated mice. We conclude from these experiments that while IL-17A is required for neutrophil recruitment to the airway in NO_2_-promoted allergic airway disease, likely through regulation of *Cxcl5*, neither IL-17A nor neutrophil recruitment to the airway is required for AHR.

**Figure 2:**
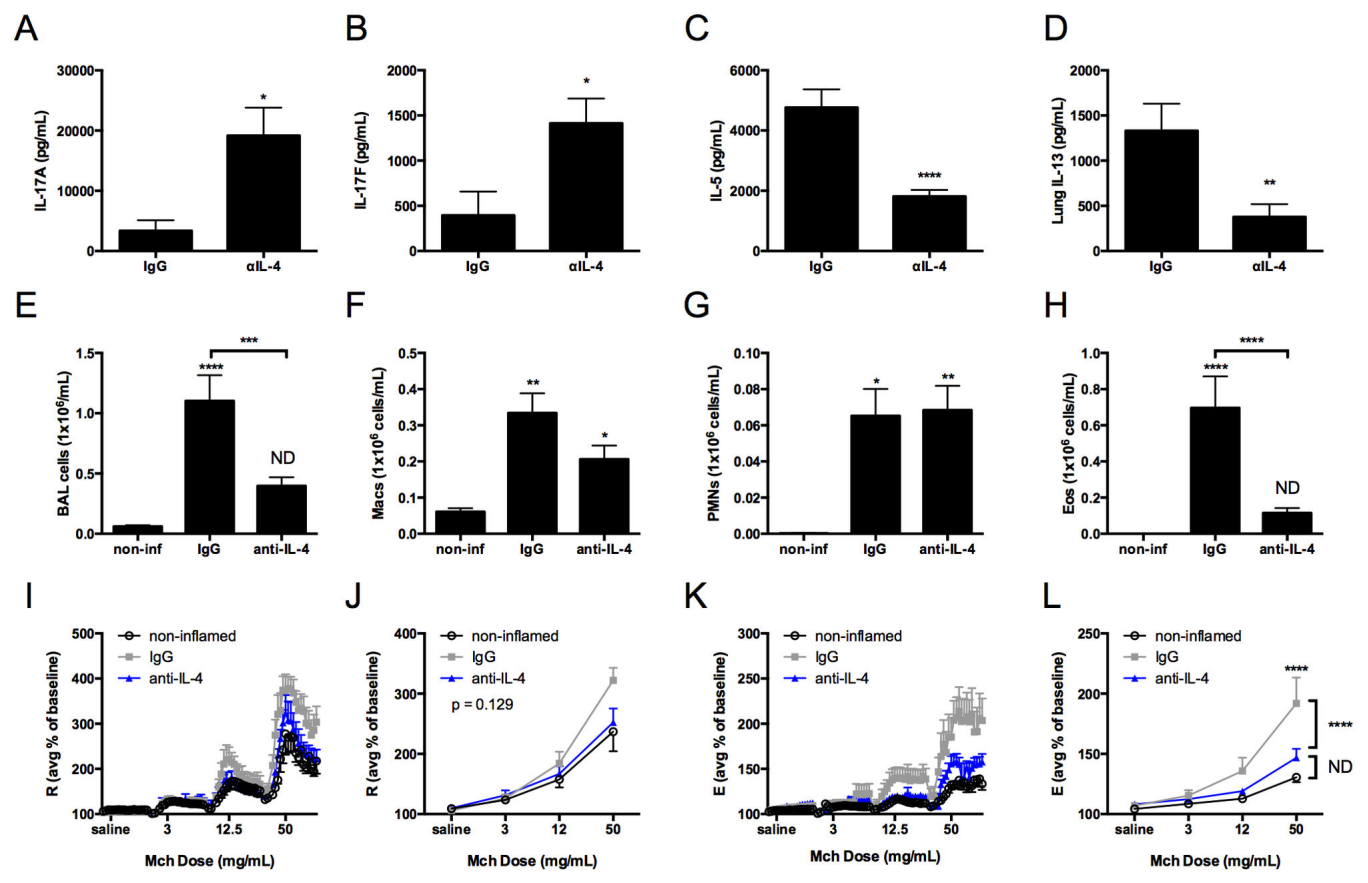
Inhibition of the Th2 response potentiates IL-17A and IL-17F production but is not sufficient to exacerbate neutrophil recruitment or AHR development. Mice were exposed to 15ppm NO_2_ for 1 hour on day 1. All mice were exposed to OVA on days 1-3 and again during challenge on days 14-16 and analyzed 48 hours after the final antigen challenge. IgG isotype control antibody was administered one day prior to NO_2_ exposure and one day prior to OVA challenge. Anti IL-4 neutralizing antibody was administered one day prior to sensitization on day 0. A second group also received anti-IL-4 antibody one day prior to OVA challenge on day 13. Because we observed no differences in AHR, BAL cell counts, or cytokine production between mice that received anti-IL-4 at sensitization only on day 0 or on day 0 and day 13, we combined anti-IL-4 treated mice for statistical analysis. 48 hours following the final antigen challenge, lungs were removed and enzymatically digested. Single cell suspensions were restimulated in the presence of 200 μg/ml OVA antigen for 96 hours prior to the harvesting of cell supernatants and analysis of cytokine production. IL-17A (A) and IL-17F (B) were quantitated by Milliplex. IL-5 (B) and IL-13 (C) were quantified by ELISA. BAL total cells (E), Macs (F), PMNs (G), and Eos (H) were determined. Percent baseline and average percent baseline per dose of methacholine were calculated for R (I-J) and E (K-L). Statistics were performed by student’s t-test (A-D), 2-way ANOVA (J and L) or 1-way ANOVA (E-H) and Tukey post hoc analysis. **** p < 0.0001, *** p < 0.001, ** p < 0.01, * p < 0.05 compared to non-inflamed unless otherwise indicated by brackets. ND, not significantly different compared to non-inflamed. n = 6-12 per group.

As others have reported IL-17A-dependent AHR and pulmonary inflammation in the absence of a Th2 response [52], we next sought to address whether IL-17A production was sufficient to promote airway inflammation and AHR in the setting of decreased Th2 responses. To address this hypothesis, we inhibited the generation of a Th2 response by administering anti-IL-4 neutralizing antibody [37,38] at the time of sensitization in NO_2_-promoted allergic airway disease. In comparison to IgG-treated mice, we quantitated increased IL-17A and IL-17F production by lung single cell suspensions restimulated in the presence of OVA antigen from mice that received anti-IL-4 neutralizing antibody prior to NO_2_ exposure (Figure 2A-B), whereas IL-5 and IL-13 were significantly diminished (Figure 2C-D). We measured no differences in IFNγ, IL-22, or GM-CSF, and IL-21 was minimally or not detected (data not shown). These data validate the inhibition of the Th2 response using anti-IL-4 neutralizing antibodies in our asthma model and demonstrate that IL-4 or a mediator controlled by IL-4 negatively regulates the production of IL-17A and IL-17F.

Treatment with anti-IL-4 neutralizing antibody resulted in a significant decrease in total BAL cells (Figure 2E) and eosinophils (Figure 2H), whereas macrophages (Figure 2F) and neutrophils (Figure 2G) were not affected. Upon assessment of MCh responsiveness, mice that received isotype control antibody, but not anti-IL-4 antibody, developed elevated resistance (trend only; Figure 2I-J) and elastance (Figure 2K-L) compared to non-inflamed mice. These data suggest that the endogenously-generated Th17 response is not sufficient to elicit AHR in NO_2_-promoted allergic airway disease. While validating our model, we discovered that to adequately assess AHR in NO_2_-promoted allergic airway disease, MCh must be administered up to a dose of 100 mg/mL since we did not observe a statistically significant increase in AHR at 50 mg/mL MCh (Figure 1E-H). However, in mice treated with IgG isotype control antibody (Figure 2L), we did observe a significant increase in AHR at 50 mg/mL MCh, suggesting that the IgG might elicit an effect independent of our asthma model. Further characterization of the impact of IgG on AHR is presented in the (Figure S1). Taken together, these data demonstrate that in the absence of a strong Th2 response, the potentiated production of IL-17A and IL-17F is not sufficient to exacerbate pulmonary inflammation or AHR above that observed in NO_2_-promoted allergic airway disease.

### The absence of the IL-1R-dependent Th17 response in NO_2_-promoted allergic airway disease correlates with exacerbation of AHR

**Figure 3:**
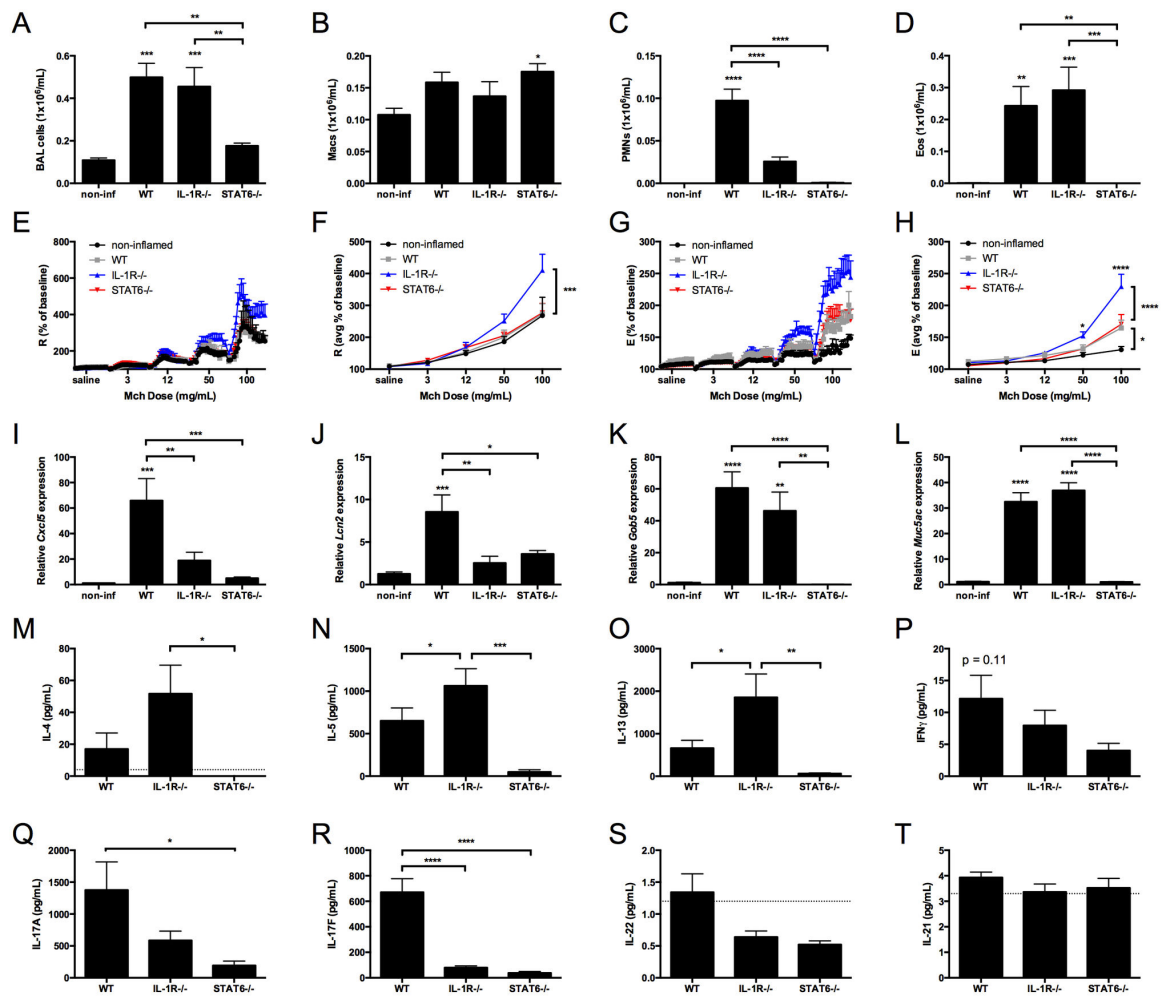
The absence of the IL-1R-dependent Th17 response in NO_2_-promoted allergic airway disease correlates with exacerbation of AHR. C57BL/6 (WT), IL-1R-/-, and STAT6-/- mice were subjected to NO_2_-promoted allergic sensitization, challenged, and analyzed 48 hours following the final antigen challenge. BAL total cells were quantified (A) and Macs (B), PMNs (C), and Eos (D) were calculated based on cell fractions. Methacholine responsiveness was performed by invasive forced oscillation technique. The percent baseline and average percent baseline per dose of methacholine were calculated for R (E-F) and E (G-H), as determined by the single-compartment model [47]. Quantitative RT-PCR was performed to measure gene expression for *Cxcl5* (I)*, Lcn2* (J), *Gob5* (K), and *Muc5ac* (L). Th2 cytokines IL-4 (M), IL-5 (N), and IL-13 (O), the Th1 cytokine IFNγ (P), and the Th17 cytokines IL-17A (Q), IL-17F (R), IL-22 (S), and IL-21 (T) were quantified by Milliplex. The dashed line in M, S, and T signifies the lower limit of detection in the assay. Statistics were performed by 1-way ANOVA (A-D and I-T) or 2-way ANOVA (F and G) and Tukey post-hoc analysis. **** p < 0.0001, *** p < 0.001, ** p < 0.01, * p < 0.05 compared to non-inflamed unless otherwise indicated by brackets. n = 5-8/group.

Th17 cells produce multiple cytokines that are known to regulate inflammation, including IL-17A, IL-17F, IL-22, and GM-CSF [28,53-55]. We have previously demonstrated a critical role for IL-1R signaling in the generation of the Th17 response, but not the Th2 response, in NO_2_-promoted allergic airway disease [11]. Because neutralization of IL-17A at antigen challenge does not protect mice against the development of AHR (Figure 1), and the potentiation of IL-17A and IL-17F production in the setting of a diminished Th2 response is not sufficient to exacerbate AHR, we hypothesized that the Th17 response contributes minimally to the airway inflammation and AHR development in our allergic asthma model. To address the role of the IL-1R-dependent Th17 response, we subjected C57BL/6 (WT) mice and IL-1R-/- mice to NO_2_-promoted allergic sensitization and antigen challenge. We also hypothesized that the Th2 immune response is required for the generation of AHR following antigen challenge. To address this hypothesis, we also subjected STAT6-/- mice, which are unable to respond to IL-4 or IL-13, to NO_2_-promoted allergic sensitization and antigen challenge [39]. Our analysis revealed that total BAL cell counts increased in WT and IL-1R-/- mice in comparison with non-inflamed control mice and STAT6-/- mice (Figure 3A). Whereas neutrophils and eosinophils were elevated in the BAL of WT mice (Figure 3C-D), neutrophils in the BAL from IL-1R-/- mice were significantly decreased compared to WT mice (Figure 3C), while numbers of eosinophils from IL-1R-/- mice were not significantly different from WT (Figure 3D). Neutrophils and eosinophils in the BAL of STAT6-/- mice were not statistically different from non-inflamed mice (Figure 3C-D). Upon assessment of MCh responsiveness, WT mice demonstrated a significant increase in elastance, but not resistance, compared to non-inflamed control mice (Figure 3E-H). Surprisingly, IL-1R-/- mice developed significantly elevated resistance and elastance above that of WT mice, whereas STAT6-/- mice developed AHR that was not statistically different from WT mice (Figure 3E-H). These data demonstrate that neither the IL-1R-dependent Th17 response nor the STAT6-dependent Th2 response is required for the development of AHR in NO_2_-promoted allergic airway disease and suggest that IL-1R signaling is protective against AHR development in this asthma model.

To further address the mechanisms that may contribute to increased AHR in IL-1R-/- mice following antigen challenge in NO_2_-promoted allergic airway disease, we quantified gene expression in whole lungs and cytokine production following OVA restimulation of lung single cell suspensions from WT, IL-1R-/-, and STAT6-/- mice. We found that the neutrophil-activating chemokine *Cxcl5*, as well as *Lcn2*, were decreased in both IL-1R-/- mice and STAT6-/- mice (Figure 3I and J). The mucin-associated genes, *Gob5* and *Muc5ac*, however, were not statistically different between WT and IL-1R-/- mice, but were decreased in STAT6-/- mice (Figure 3K and L). Following the restimulation of lung single cell suspensions in the presence of antigen, the production of Th2 cytokines IL-4, IL-5, and IL-13 were increased in IL-1R-/- mice compared to WT or STAT6-/- mice (Figure 3M-O). In contrast, IL-17A and IL-17F production were decreased in IL-1R-/- mice and STAT6-/- mice compared to WT mice (Figure 3Q-R). A similar trend existed for IFNγ (Figure 3P) and minimal amounts of IL-21 and IL-22 were detected (Figure 3S-T). Overall, these data suggest that the IL-1R/Th17 axis may inhibit the Th2 adaptive immune response in NO_2_-promoted allergic airway disease at the time of antigen challenge.

### IL-17A production by lung cells from NO_2_-allergically sensitized mice is qualitatively different from that of Th17-transferred mice

The adoptive transfer of *in vitro* polarized effector T-cells is a method for investigating the *in vivo* consequences of an antigen-specific immune response [32]. The observation that IL-17A, neutrophils, and the Th17 response do not contribute to AHR in our asthma model clearly contrasts with that observed from a model in which adoptive transfer of *in vitro* polarized Th17 OTII T-cells is followed by antigen challenge [32]. To better understand the Th17 response generated endogenously in NO_2_-promoted allergic airway disease, we compared it to the responses generated following adoptive transfer of antigen-specific Th17 or Th2 cells followed by OVA challenge.

**Figure 4:**
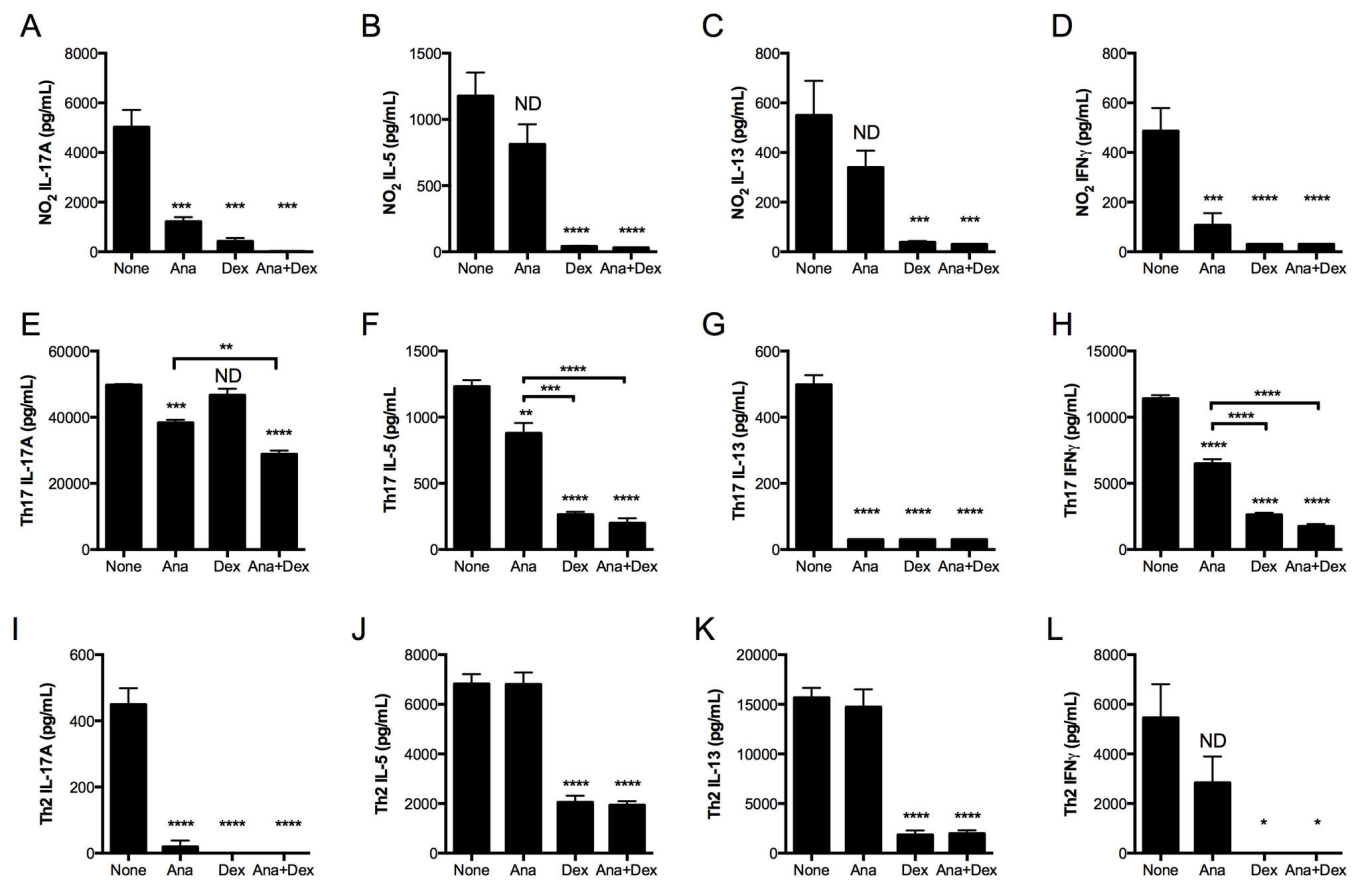
IL-17A production by lung cells from NO_2_-promoted and allergically sensitized mice is qualitatively different from IL-17A production by lung cells following Th17 adoptive transfer and antigen challenge. Mice were subjected to NO_2_-promoted allergic sensitization, OVA-challenged, and analyzed 48 hours after the final antigen challenge. For adoptive transfer, CD4^+^ T-cells from OTII splenocytes by were either Th2 or Th17 polarized *in* *vitro* and adoptively transferred to recipient mice, which were then OVA-challenged for 3 consecutive days and analyzed 24 hours following the final OVA challenge. At analysis, lungs were removed and enzymatically digested. Lung single cell suspensions were restimulated with OVA antigen in the presence or absence of anakinra (Ana; 0.2 μg/mL), Dex (10^-8^ M), or anakinra and Dex in combination for 96 hours. Cell supernatants from antigen-restimulated and treated lung cells from mice subjected to NO_2_-promoted allergic sensitization and OVA challenge (A-D), Th17 adoptive transfer (E-H), or Th2 adoptive transfer (I-L) were harvested and cytokine levels were quantitated by ELISA. Statistics were performed by one-way ANOVA and Bonferroni post hoc analysis. **** p < 0.0001, *** p < 0.001, ** p < 0.01, * p < 0.05 compared to controls (None) unless otherwise indicated by brackets. ND, not significantly different compared to controls. For NO_2_/OVA sensitized and challenged mice, n = 6. For Th17 and Th2 adoptively transferred mice, samples from n = 3 mice were pooled prior to *in* *vitro* culturing, which was performed in triplicate. Adoptive transfer data are representative of 2 independent studies.

Single-cell suspensions of lungs from NO_2_-sensitized and challenged mice (bars labeled “None”, Figure 4A-D) produced IL-17A, IL-5, IL-13, and IFNγ upon *in vitro* restimulation, whereas recipient mice of the Th17 adoptive transfer produced 10-fold more IL-17A, similar levels of IL-5 and IL-13, and 20-fold more IFNγ (bars labeled “None”, Figure 4E-H). These data indicate that the endogenously-generated Th17 response in NO_2_-promoted allergic airway disease is of decreased magnitude compared to that generated following adoptive transfer of *in vitro* polarized Th17 OTII T-cells. Restimulated lung cells from Th2-transferred mice produced much higher levels of the Th2 cytokines IL-5 and IL-13 than either NO_2_-allergically inflamed mice or mice that had received Th17 cells, whereas IL-17A production was 10-fold less than that of the NO_2_-allergically inflamed mice and 100-fold less than Th17 adoptively transferred mice, and IFNγ production was 10-fold greater than that of the NO_2_-allergically inflamed mice and 2-fold less than Th17 adoptively transferred mice (bars labeled “None”, Figure 4I-L).

The Th17 response generated upon adoptive transfer results in GC-resistant allergic airway disease, whereas the Th2 adoptive transfer is GC-sensitive [32]. Since IL-17A production and neutrophils, which are thought to promote resistance to GC treatment [30,56], are present in our model following antigen challenge, we sought to contrast the relative GC sensitivity of the Th17 response in NO_2_-promoted allergic airway disease to that in the Th17 and Th2 adoptive transfer models. The *in vitro* polarization of Th17 T-cells does not require IL-1R signaling [19], which contrasts with the IL-1R-dependent Th17 response in NO_2_-promoted allergic airway disease [11]. Furthermore, IL-1R signaling is an attractive therapeutic target in the treatment of clinical asthma [57-59]. However, it remains unknown whether the Th17 immune response generated at antigen challenge, a clinically relevant time point, requires IL-1R signaling. We applied an *ex vivo* approach to address the GC sensitivity of the endogenously-generated Th17 response at antigen challenge and to test whether the antigen-specific Th17 response in the lung requires IL-1R signaling. Following antigen challenge in either NO_2_-promoted allergic sensitization or the adoptive transfer of *in vitro* polarized Th17 or Th2 OTII T-cells and antigen challenge, we incubated lung single-cell suspensions with the IL-1 receptor antagonist anakinra, the GC dexamethasone (Dex), the combination, or no inhibitors in the presence of OVA antigen. Sensitivity to either Dex or anakinra was defined as ≥50% decrease in cytokine production compared to lung cells from similarly-treated mice incubated in the presence of OVA alone. Synergistic inhibition by the combination of anakinra and Dex was defined as ≥50% decrease in cytokine production compared with lung cells from similarly treated mice that were incubated with OVA and either Dex or anakinra.

In NO_2_-promoted allergic airway disease, IL-17A production by OVA-restimulated lung single cell suspensions was almost completely inhibited by either anakinra or Dex treatment, and the combination completely inhibited production of IL-17A (Figure 4A). In contrast, lung cell production of the Th2 cytokines IL-5 and IL-13 was resistant to anakinra treatment (Figure 4B-C). Dex treatment completely inhibited Th2 cytokine production, which was not further diminished by the addition of anakinra (Figure 4B-C). The Th1 cytokine IFNγ was inhibited by either Dex or anakinra treatment (Figure 4D). IL-17A production by antigen-restimulated lung cells generated from recipients of *in vitro* polarized Th17 cells decreased significantly but not ≥50% in the presence of anakinra but not Dex. The combination of Dex and anakinra further decreased IL-17A production, although the levels remained ≥50% of cytokine production by OVA-stimulated lung cells in the absence of inhibitors (Figure 4E). Production of IL-5 and IFNγ were significantly decreased when lung cells from Th17-transferred mice were restimulated with OVA in the presence of anakinra, although this decrease was less than 50% of the IL-5 or IFNγ production by lung cells restimulated in the presence of OVA alone (Figure 4F, 4H). IL-5 and IFNγ production by lung cells from recipients of Th17 cells were sensitive to Dex treatment (Figure 4F, 4H), whereas IL-13 was sensitive to both anakinra and Dex (Figure 4G). We conclude that endogenous IL-17A production in NO_2_-promoted allergic airway disease is sensitive to both anakinra and Dex, whereas Th2 cytokines IL-5 and IL-13 are sensitive to Dex but not anakinra (results summarized in Table 1). In contrast, Th17 adoptive transfer results in IL-17A production that is resistant to Dex, anakinra, or the combination of anakinra and Dex, and Th2 cytokine production that demonstrates Dex sensitivity with variable sensitivity to anakinra that is cytokine-dependent.

**Table 1:** Summary of anakinra and dexamethasone sensitivity profiles.

	**NO_2_-promoted allergic airway disease**			**Th17 adoptive transfer**			**Th2 adoptive transfer**			**Anti-IL-4 in NO_2_-promoted allergic airway disease**		
**Cytokine**	**Ana**	**Dex**	Ana+ Dex*	**Ana**	**Dex**	Ana+ Dex*	**Ana**	**Dex**	Ana+ Dex*	**Ana**	**Dex**	Ana+ Dex*
**IL-17A**	S	S	S	R	R	S	S	S	R	S	S	S
**IL-17F**	S	S	S	R	R	S	NM	NM	NM	NM	NM	NM
**IL-21**	ND	ND	ND	R	R	R	NM	NM	NM	NM	NM	NM
**IL-22**	S	S	S	R	R	S	NM	NM	NM	NM	NM	NM
**GM-CSF**	S	S	R	S	S	R	NM	NM	NM	NM	NM	NM
**IL-5**	R	S	R	R	S	R	R	S	R	R	S	R
**IL-13**	R	S	R	S	S	R	R	S	R	R	S	R
**IFNγ**	S	S	R	R	S	R	R	S	R	S	S	S

S, sensitive; R, resistant; NM, not measured; ND, not detected GM-CSF, granulocyte-macrophage colony-stimulating factor.

Sensitivity to dexamethasone (Dex) or anakinra (Ana) is defined as ≥ 50% decrease in cytokine production in comparison to the appropriate antigen-restimulated control.

* Cases in which the cytokine is Dex sensitive, addition of anakinra further decreased the production by ≥ 50%.

It is possible that the relative resistance of IL-17A production to *in vitro* treatment with Dex or anakinra is a property of polarization in an *in vitro* system, and that established effector IL-17A-producing cells are resistant to inhibition by Dex, similar to that hypothesized by others [60]. Notably, we also observed IL-17A production by antigen-restimulated lung cells from recipient mice of *in vitro* polarized OTII Th2 cells following antigen challenge (Figure 4I). We therefore tested the *in vitro* Dex or anakinra sensitivity of OVA-restimulated lung cells from mice that received *in vitro* polarized OTII Th2 cells. Similar to that observed in NO_2_-allergically inflamed mice, but distinct from that observed in Th17 adoptively transferred mice, IL-17A production by lung cells from Th2-transferred mice was almost completely inhibited in the presence of anakinra or Dex (Figure 4I). Production of the Th2 cytokines IL-5 and IL-13, as well as the Th1 cytokine IFNγ, were resistant to treatment with anakinra, but susceptible to inhibition by Dex (Figure 4J-L). Thus, the *in vitro* treatment of lung cells from recipients of the Th2 adoptive transfer exhibited anakinra and Dex sensitivity similar to that of the NO_2_-promoted, endogenously generated immune response, which is in contrast to the resistance of IL-17A production to treatment with Dex and anakinra from recipients of the Th17 adoptive transfer. These results indicate that there are consequences of *in vitro* polarization or characteristics of OTII cells that do not reflect the observed resistance of IL-17A production to anakinra or Dex treatment during antigen-restimulation of lung cells from Th17 adoptively-transferred mice.

### Inhibition of the Th2 response does not confer anakinra- or Dex-resistant IL-17A production in NO_2_-promoted allergic airway disease

**Figure 5:**
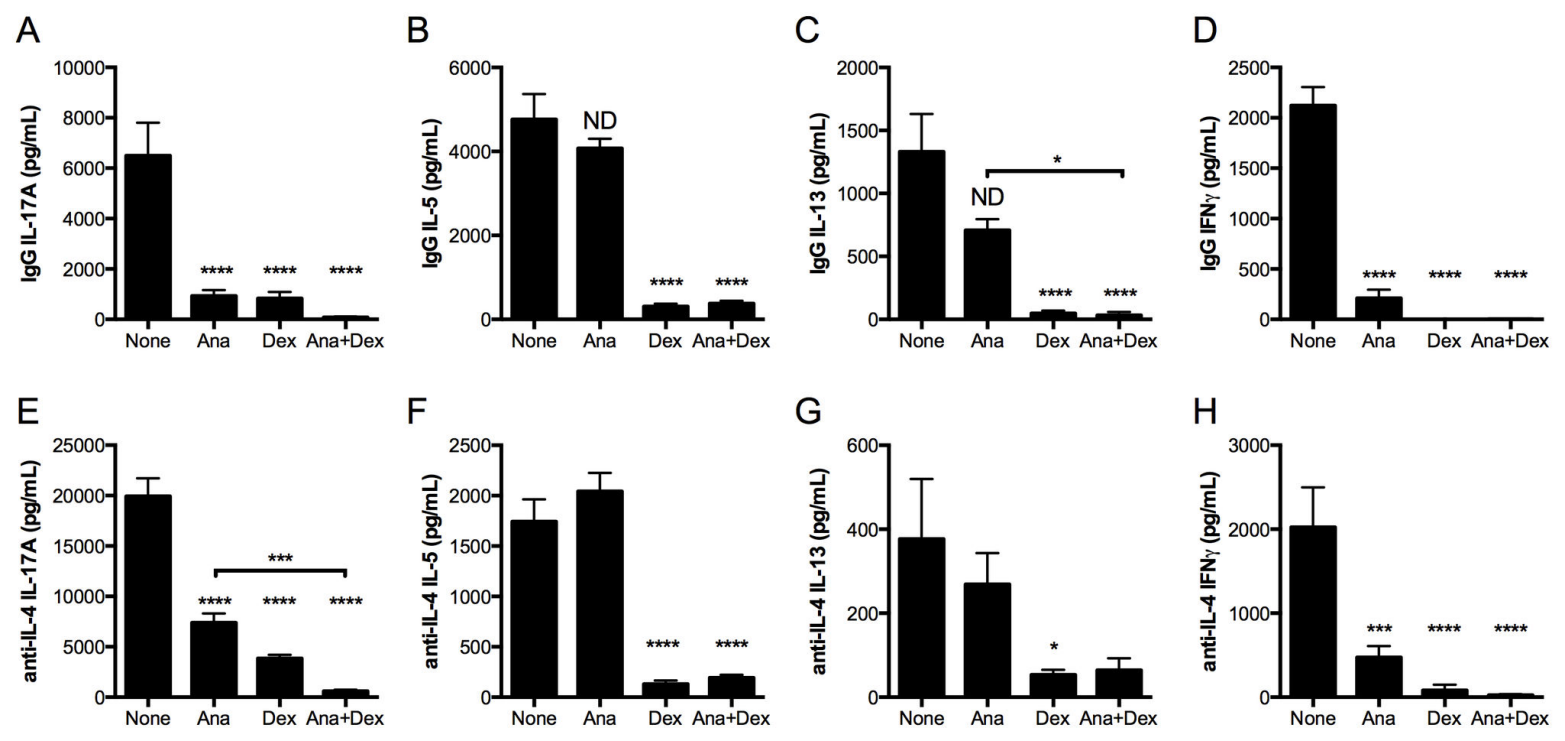
Inhibition of the Th2 response with anti-IL-4 antibodies does not confer anakinra or Dex resistance in IL-17A production by antigen-restimulated lung cells in NO_2_-promoted allergic airway disease. Mice were treated as in Figure 2. Lung single cell suspensions were restimulated with OVA antigen in the presence or absence of anakinra (Ana; 1 μg/mL), Dex (10^-8^ M), or anakinra and Dex in combination for 96 hours. Cell supernatants from antigen-restimulated and treated lung cells from IgG isotype control treated mice (A-D) or anti-IL-4 treated mice (E-H) were analyzed for cytokines by ELISA. Statistics were performed by one-way ANOVA and Bonferroni post hoc analysis (E-L). **** p < 0.0001, *** p < 0.001, ** p < 0.01, * p < 0.05 compared to controls (none) unless otherwise indicated by brackets. ND, not significantly different compared to controls. n = 6-12 per group.

In addition to IL-17A, the Th2 cytokine IL-4 is capable of regulating the expression of the GC receptor and may impact GC responsiveness [59]. Since the presence of a Th2 response can dampen IL-17A production (Figure 2A and E [52]), we reasoned that the relative absence of the Th2 response in the Th17 adoptive transfer model compared with that generated during NO_2_-promoted allergic airway disease (Figure 4B-C versus 4F-G) may explain the differences in sensitivity to treatment with Dex and anakinra during *in vitro* restimulation. Therefore, we administered anti-IL-4 or IgG isotype control antibody prior to NO_2_ sensitization and antigen challenge, as in Figure 2. *In vivo* administration was followed by the pharmacologic treatment of lung cells restimulated in the presence of OVA antigen with either anakinra, Dex, or the combination of anakinra and Dex. The anakinra dose used in this experiment was 1 μg/mL, which was higher than that used for the studies presented in Figure 4. However, incubation with increasing doses of anakinra did not further inhibit IL-17A or IL-5 production by lung cells from recipient mice of the Th17 adoptive transfer during antigen-restimulation (Figure S2). In both IgG and anti-IL-4 treated mice, IL-17A levels were significantly inhibited with either Dex or anakinra (Figure 5A and E). In addition, IL-17A was further decreased by the combination of anakinra and Dex in anti-IL-4 treated mice. In both IgG and anti-IL-4 treated mice, IL-5 and IL-13 production were unaffected by *in vitro* treatment with anakinra, whereas Dex significantly inhibited the production of these Th2 cytokines (Figure 5B-C and Figure 5F-G). IFNγ levels in both IgG and anti-IL-4 treated mice decreased in the presence of either anakinra or Dex (Figure 5D and Figure 5H). Thus, the endogenously-generated Th17 response remained sensitive to both anakinra and Dex treatment when IL-4 was neutralized *in vivo*. The residual Th2 response in anti-IL-4 treated mice remained sensitive to Dex treatment during *in vitro* restimulation of lung cells in the presence of OVA antigen (summarized in Table 1). Thus, the potentiated production of IL-17A and IL-17F during Th2 inhibition exhibits similar Dex and anakinra sensitivity as IgG control antibody treated mice. We therefore conclude that Th2 interactions with the Th17 response do not impact the Dex/anakinra sensitivity profile.

### The endogenously-generated Th17 response in NO_2_-promoted allergic airway disease is distinct from the Th17 response generated following Th17 adoptive transfer

**Figure 6:**
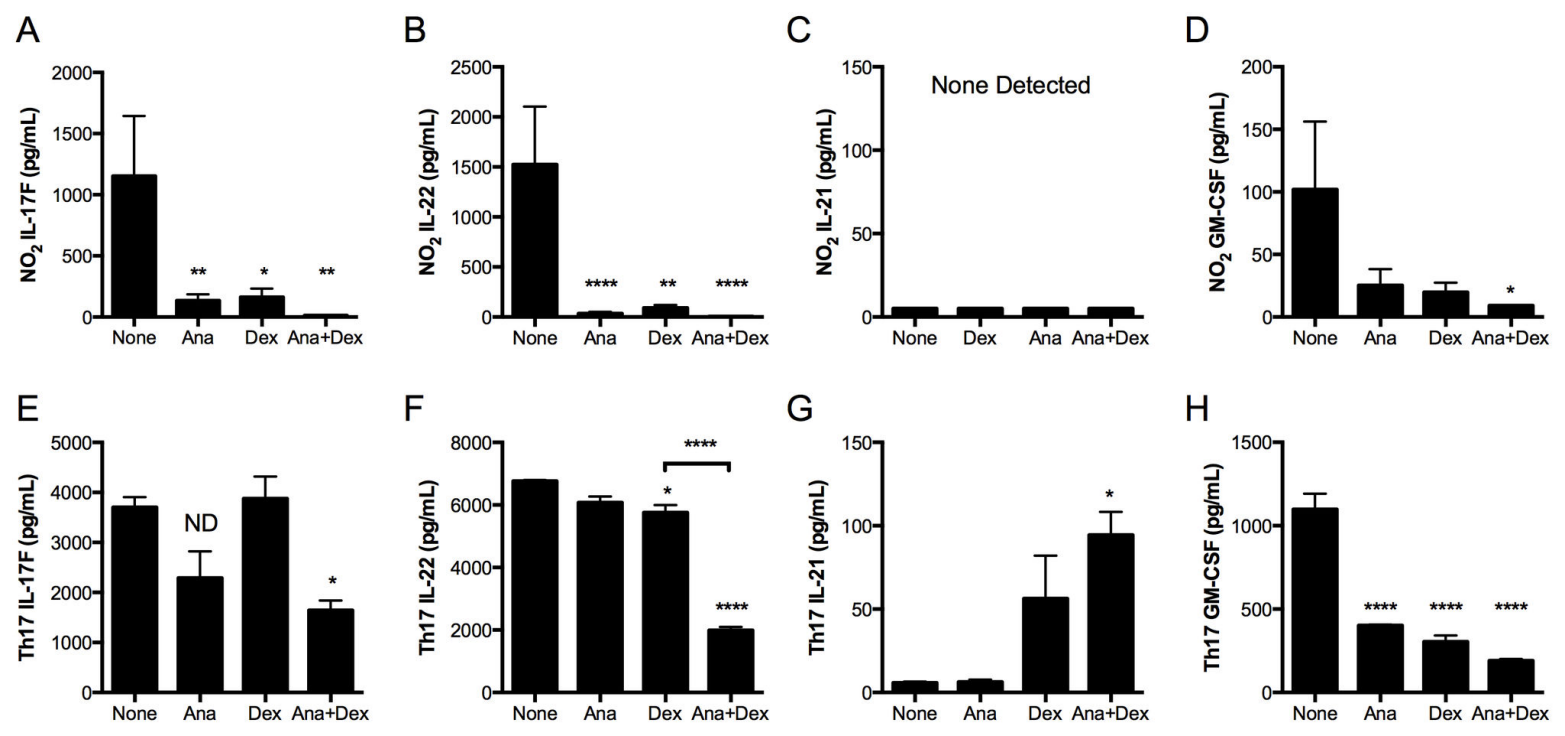
The endogenously-generated Th17 response in NO_2_-promoted allergic airway disease is qualitatively different from the Th17 response generated following Th17 adoptive transfer. Mice were treated as in Figure 4. Lung single-cell suspensions were restimulated with OVA antigen in the presence or absence of anakinra (Ana; 0.2 μg/mL), Dex (10^-8^ M), or anakinra and Dex in combination for 96 hours. Cell supernatants of antigen-restimulated and treated lung cells from mice subjected to NO_2_-promoted allergic sensitization and OVA challenge (A-D) or Th17 adoptive transfer (E-H) were analyzed for the production of cytokines by Milliplex. Statistics were performed by 1-way ANOVA and Bonferroni post hoc analysis. **** p < 0.0001, *** p < 0.001, ** p < 0.01, * p < 0.05 compared to controls (None) unless otherwise indicated by brackets. ND, not significantly different compared to controls. For NO_2_/OVA sensitized and challenged mice, n = 6. For Th17 and Th2 adoptively transferred mice, samples from n = 3 mice were pooled prior to *in* *vitro* culturing, which was performed in triplicate.

We next addressed whether the broader Th17 response manifest in NO_2_-promoted allergic airway disease and Th17 adoptive transfer demonstrated the qualitative differences in Dex and anakinra sensitivity exhibited by the production of IL-17A (Figure 4A and 4E). Therefore, we quantified Th17 cytokine production by lung cells restimulated with OVA antigen in the presence or absence of anakinra and Dex treatment following NO_2_-promoted sensitization and antigen challenge (Figure 6A-D) or the Th17 adoptive transfer and antigen challenge (Figure 6E-H). Lung cells restimulated in the presence of antigen and either anakinra or Dex from NO_2_-allergically inflamed mice produced less IL-17F (Figure 5A), IL-22 (Figure 5B), and GM-CSF (trend only; Figure 5D) than when restimulated in the absence of inhibitors. No IL-21 was detected from restimulated lung cells in NO_2_-promoted allergic airway disease (Figure 5C). The diminution of IL-17F, IL-22, and GM-CSF was greater than 50% for either Dex or anakinra treatment, which was further diminished with the combination of anakinra and Dex. Therefore, Th17 cytokines from NO_2_-allergically inflamed mouse lungs are sensitive to both anakinra and Dex treatment *in vitro* (results summarized in Table 1).

For lung single-cell suspensions from Th17-adoptively transferred mice, the production of IL-17F was not affected by Dex, and a statistically significant decrease was observed for the combined treatment of Dex and anakinra (Figure 6E). Despite this decrease the levels of IL-17F remained greater than 50% of IL-17F production from lung cells stimulated with OVA. A slight decrease in IL-22 was observed with Dex treatment, which was substantially potentiated and achieved ≥50% reduction by the addition of anakinra (Figure 5F), whereas IL-22 production by lung cells treated with Dex alone remained ≥50% of the IL-22 production by otherwise untreated OVA-restimulated lung cells. No difference for IL-17F or IL-22 was observed upon *in vitro* treatment with anakinra (Figure 5E-F). Similar to that observed in the NO_2_ allergically inflamed mice, IL-21 was not detected in lung cells restimulated in the presence of OVA alone (Figure 6G). However, in *vitro* treatment with Dex during antigen restimulation of lung cells from recipients of the Th17 adoptive transfer resulted in increased IL-21 production (trend only), and a statistically significant increase in IL-21 production with the combination of Dex and anakinra (Figure 5G). The production of GM-CSF by lung cells from Th17-transferred mice decreased upon *in vitro* treatment with Dex or anakinra (Figure 6H). In summary, production of IL-17F (Figure 5E) and IL-22 (Figure 5F) by antigen-restimulated lung cells from Th17 adoptively transferred mice are resistant to treatment with either Dex or anakinra alone. These cytokines exhibit the same Dex and anakinra resistance as IL-17A production by lung cells from Th17 adoptive transfer mice (Figure 3E). In contrast, GM-CSF is inhibited by Dex and anakinra treatment. Interestingly, Dex or the combination of Dex and anakinra induce the production of IL-21. In comparison, the endogenously-generated Th17 response in NO_2_-promoted allergic airway disease is sensitive to *in vitro* treatment with anakinra or Dex. These results demonstrate that cytokine production by the endogenously-generated Th17 response in NO_2_-promoted allergic airway disease is sensitive to Dex and IL-1R-dependent during *ex vivo* antigen restimulation, whereas the Th17 immune response following the adoptive transfer of *in vitro* polarized Th17 cells is Dex resistant and independent of IL-1R signaling. Therefore, we conclude that the endogenously generated Th17 response in NO_2_-promoted allergic airway disease is qualitatively distinct from that generated following Th17 adoptive transfer and antigen challenge.

## Discussion

The Th17 response has been implicated in promoting glucocricoid-resistant asthma and allergic airway disease [16,17,32]. Clinical data also support a role for IL-1R signaling in asthma [14,61-64]. As such, the IL-1R-Th17 axis is an attractive therapeutic target for study in animal models of allergic airway disease and, more importantly, in human asthma [57,65]. Here we investigated the functional role of the IL-1R-dependent Th17 response in NO_2_-promoted allergic airway disease, demonstrating that while IL-17A and IL-1R are critical in neutrophil recruitment, both are dispensable for promoting AHR. Furthermore, our data indicate that IL-1R is protective in AHR development and associates with diminished Th2 responses in NO_2_-promoted allergic airway disease. These results illustrate a very different function of the endogenously-generated Th17 response in NO_2_-promoted allergic airway disease than that observed following the adoptive transfer of *in vitro* polarized Th17 cells. We observed in the NO_2_ model that Th17 cytokine production by lung single cell suspensions restimulated in the presence of OVA antigen was sensitive to inhibition by either anakinra or Dex treatment. In contrast, lung cells from mice that received *in vitro* polarized Th17 OTII T-cells upon adoptive transfer and were subsequently challenged with OVA demonstrated resistance to treatment with anakinra or Dex. This qualitative difference is not entirely explained by differences in magnitude or by the absence of a Th2 response in the adoptive transfer model. Taken together, our data support a nonpathogenic and potentially protective role for the endogenously-generated Th17 adaptive immune response in NO_2_-promoted allergic airway disease, which is IL-1R-dependent and sensitive to inhibition by Dex treatment during antigen restimulation *ex vivo*. Furthermore, these results demonstrate that Th17 adoptive transfer does not recapitulate the Th17 response generated endogenously in NO_2_-promoted allergic airway disease. The mechanisms that govern these differences remain to be determined.

IL-17A is thought to promote airway inflammation by inducing the production of chemokines by epithelial cells, endothelial cells, smooth muscle cells, and fibroblasts [15,55,66]. Furthermore, in allergic airway disease, IL-17A is the Th17 cytokine implicated in AHR [13,28,32] and airway remodeling [49]. Th17-mediated allergic airway disease acts through IL-17A/IL-17R signaling to recruit neutrophils, and both IL-17A and neutrophils are required to promote AHR development [13,18,32]. Therefore, we sought to address specifically whether IL-17A was required for AHR in our model of NO_2_-promoted allergic airway disease.

Similar to our previous reports [9,11,12], we clearly observed mixed Th2/Th17 airway inflammation, including the recruitment of neutrophils and eosinophils, in NO_2_-promoted allergic airway disease. Neutrophil recruitment required IL-17A, likely through the induction of *Cxcl5* by pulmonary epithelial cells [67], validating that IL-17A is a critical contributor to airway neutrophila in NO_2_-promoted allergic airway disease [68]. However, contrary to our hypothesis, treatment with an IL-17A-neutralizing antibody did not impact the development of MCh hyperresponsiveness compared to untreated NO_2_-sensitized and challenged mice and resulted in increased elastance compared to mice administered and isotype control antibody. We have previously noted a trend for decreased neutrophils in IL-1R-/- mice [11], and in these studies, we observed a significant diminution of neutrophils in NO_2_-promoted allergic airway disease following antigen challenge. Yet surprisingly, IL-1R-/- mice developed exacerbated AHR compared with WT mice.

Neutrophil activity is thought to mediate asthma by inducing structural damage via the release of enzymes from granules [33,69]. In a model of alum/OVA allergic airway disease, inhibition of neutrophil elastase prevented AHR, mucus production, eosinophil recruitment to the airway, and Th2 cytokine production, as well as eotaxin in the BAL, supporting a role for neutrophil elastase in the generation of Th2 responses as well [70]. In contrast, a study conducted in an alum/OVA model demonstrated that the inhibition of neutrophils by blocking IL-1R signaling early during antigen challenge did not impact the development of AHR [71]. We also observed that inhibition of neutrophil recruitment with either anti-IL-17A neutralizing antibody, or through genetic deficiency in IL-1R, provided no protection against AHR in our asthma model. Thus, our results do not support a role for IL-17A or neutrophil recruitment in the development of AHR in NO_2_-promoted allergic sensitization.

We have not detected elastase activity at the time of analysis in either BAL cells or BAL fluid, although incubating BAL cells for 24h at RT resulted in elastase activity (data not shown), suggesting that the neutrophils recruited to the lavageable airspaces in our model at the time of analysis are inactive. Furthermore, we have conducted antibody-mediated neutrophil depletion at the time of antigen challenge in our model and found substantial variability in its effect on AHR.

The involvement of the IL-1R in promoting Th17 responses that then regulate AHR development have not to our knowledge been published. Toluene diisocyanate-mediated AHR and mucus production required IL-1R signaling, but IL-17 was not measured in this model [72]. In HDM-promoted allergic airway disease and an alum-independent model involving intraperitoneal OVA injection, IL-1R was required for the development of a Th2 response [53,73]. In NO_2_-promoted allergic airway disease, the IL-1R is critical for the Th17 response but not the Th2 response [11]. Whereas the IL-1R-dependent Th17 response has been implicated in experimental allergic encephalomyelitis (EAE) susceptibility [23], its importance has also been a subject of debate since IL-17A-/-, IL-17F-/-, and IL-17A overexpressing mice were equally susceptible to develop EAE and had similar disease severity, whereas minimal protection was elicited by anti-IL-17A treatment of IL-17F-/- mice [74]. Absence of a role for the Th17 response was also recently identified in an alum/OVA model of allergic airway disease [75]. In agreement with these findings, our results demonstrate that neutralization of IL-17A at the time of challenge clearly does not prevent the induction of AHR in NO_2_-promoted allergic airway disease.

In a number of studies, IL-17A was found to be protective in the setting of allergic airway disease. In an alum/OVA model, administration of IL-17A at the time of challenge decreased AHR, eosinophils in the BAL, as well as cellular infiltrate and mucus on histology, whereas anti-IL-17A exacerbated these parameters [26]. In a model of RSV-promoted asthma exacerbation, IL-17A-/- mice exhibited increased AHR despite a decrease in neutrophils, demonstrating a protective role for IL-17A [25]. In these models, IL-17A acts as a negative regulator of the Th2 immune response, resulting in decreased inflammation and diminished AHR. Administration of anti-IL-17A neutralizing antibody had no effect on eosinophil recruitment or Th2 cytokine responses on antigen restimulation of lung cells in our model of NO_2_-promoted allergic airway disease. While we observed a trend for increased *Gob5* expression in the lungs of mice that had been treated with anti-IL-17A antibody prior to antigen challenge, we did not observe the same trend in IL-1R-/- mice. Thus, IL-17A does not influence Th2 responses in our model. In contrast, IL-1R-/- mice developed exacerbated AHR compared to WT mice, suggesting a protective role for the IL-1R-dependent Th17 response in NO_2_-promoted allergic airway disease. Interestingly, while we observed no differences in eosinophil recruitment or the expression of mucin-associated genes, lung cells from IL-1R-/- produced elevated levels of Th2 cytokines following restimulation in the presence for OVA antigen. IL-13 is sufficient to elicit AHR [24] and may be the cytokine responsible for exacerbation of this endpoint in IL-1R-/- mice in NO_2_-promoted allergic airway disease.

Previous studies support the prevailing notion that either the Th2 or the Th17 immune response is sufficient to drive pulmonary inflammation and AHR in allergic airway disease [18,32]. For example, IL-4/IL-13 double knockout mice exhibit IL-4-dependent exaggerated IL-17 production, and despite decreased eosinophils and mucus production, these mice developed AHR similar to WT mice, which could be inhibited by administration of an anti-IL-17A antibody [52]. Such findings prompted us to test the hypothesis that the Th17 response in the absence of a Th2 response is sufficient to elicit pulmonary inflammation and AHR in NO_2_-promoted allergic airway disease. Following antigen challenge, we also observed increased IL-17A and IL-17F, but not IL-22, production by lung cells from anti-IL-4 treated mice restimulated in the presence of antigen. This is an important distinction since IL-17A and IL-17F can function redundantly to promote inflammation, whereas IL-22 can negatively regulate inflammation in allergic airway disease [54,76]. Thus, inhibition of the Th2 response by the administration of anti-IL-4 neutralizing antibody upregulates the inflammatory cytokines thought to mediate inflammation, while not impacting the production of the anti-inflammatory cytokine IL-22 [54,76]. AHR in anti-IL-4 treated mice is not statistically different from non-inflamed mice, whereas IgG treated mice exhibit robust AHR responses above that of non-inflamed and anti-IL-4 treated mice. These data indicate that the Th17 response is not sufficient to drive AHR. However, in control experiments, we also noted that administration of IgG isotype control antibody at sensitization in combination with NO_2_ exposure resulted in the development of more substantial AHR than that observed in otherwise untreated mice subjected to the NO_2_-promoted allergic airway disease model. Importantly, we did not observe any effect on AHR by IgG isotype control antibody when administered at the time of antigen challenge. Because of the IgG effect, however, we cannot conclude that inhibition of the Th2 response is completely protective in this model, since we do observe a small trend for increased elastance in anti-IL-4 treated mice compared to non-inflamed mice. Nonetheless, it is evident that the exaggerated production of IL-17A and IL-17F is not sufficient to exacerbate AHR above that observed in NO_2_-promoted allergic airway disease.

Because STAT6 is required for both the development and the effector function of the Th2 response [31,77], we subjected STAT6-/- mice to NO_2_-promoted allergic sensitization and antigen challenge to determine the role of the Th2 response in this model. In an LPS model of allergic sensitization, STAT6 was required for eosinophils and neutrophils in the airway and AHR following antigen challenge [13]. Similarly, we observed a complete absence of eosinophils and neutrophils in the BAL of STAT6-/- mice. As expected, we also observed diminution of Th2 cytokines following the restimulation of lung cells in the presence of OVA antigen. In contrast with the LPS sensitizing scheme, STAT6-/- mice developed comparable AHR to that of WT mice. Differences in experimental protocol may account for this discrepancy. Alternatively, STAT6 can promote a population of Treg cells, and FOXP3^+^ Treg cells from OVA-tolerized mice can undergo cell division upon restimulation in the presence of OVA [39,60]. Thus, the absence of a Treg population may explain increases in AHR in STAT6-/- mice despite evidence that effector T-cell populations are absent.

Others have demonstrated that inhibiting the IL-4R-STAT6 pathway may not be sufficient to inhibit alternative pathways capable of promoting AHR [31,78]. In a chronic inhalation model, airway inflammation and AHR in STAT6-/- mice coincided with a switch to a Th1 response [79]. However, we observed minimal IFNγ production in lung restimulation assays. RSV-induced IL-17A was able to elicit mucus metaplasia in the absence of STAT6, and *in vitro*, IL-17A was sufficient to induce mucus secretion in STAT6-/-, but not in WT, tracheal epithelial cells, suggesting that STAT6-/- is a negative regulator of Th17-promoted inflammation and that IL-17 may promote mucus production in the absence of a Th2 response [31]. Notably, these results were reported from an adoptive transfer model, which we propose may function differently than an endogenously-generated Th17 response [60]. We observed substantial attenuation of the Th17 response in STAT6-/- mice and mucin gene expression was comparable to that of non-inflamed mice. Similarly, *Cxcl5* and *Lcn2* expression were decreased, which support the absence of a Th17 response. This finding contradicts the concept that generation of a Th17 response is independent of or negatively-regulated by STAT6 [40,80]. There is evidence for Th2 cells to produce Th17 cytokines [81]. A population of memory Th2 cells in humans and mice was identified that produces IL-17 and coexpresses *GATA3* and *RORγt* [81]. Whereas adoptive transfer of IL-17^+^ Th2 cells elicited responses similar to the combined adoptive transfer of Th2 and Th17 cells, synergistically increasing inflammation [81,82], we observed no evidence for synergistic interplay of the Th17 and the Th2 response in driving NO_2_-promoted allergic airway inflammation.

In our studies, NO_2_ allergically inflamed and challenged STAT6-/- mice were essentially absent of airway inflammation, despite the development of AHR. Others have also observed the uncoupling of the immune response with AHR [13,77,83]. In these studies, we observed that gene expression of defensins (*Defb1*, *Defb2*, *Defb3*, *Defb4*), *A20, Nox4*, *S100a9*, *Brp-39*, *Chia* did not correlate with AHR development following NO_2_-promoted allergic airway disease (data not shown). We have previously reported *Saa3* as a candidate mediator of Th17 response induction [41] and AHR exacerbation [5]. While we observed an increase in *Saa3* expression following antigen challenge in NO_2_-promoted allergic airway disease, we observed a significant diminution of *Saa3* expression in allergically sensitized and challenged IL-1R-/- mice compared to WT mice, indicating that *Saa3* expression is not required for AHR development and suggesting that SAA3 was not responsible for exacerbated AHR in IL-1R-/- mice. We have previously reported that activation in the airway epithelium of the transcription factor NF-κB is not required for the development of AHR despite potently modulating airway inflammation [42]. These results suggest that mechanisms driving AHR exist that are independent of conventional immune-mediated inflammation. Although not studied by us, alterations in neural signaling provide another potential non-immune regulatory system controlling smooth muscle contractility [84-86]. In addition, certain mouse strains have been demonstrated to be more appropriate for measurements of AHR, particularly in response to IL-17A, and C57BL/6 mice are amongst the poorest strains in which to study this response [43,87].

The adoptive transfer of *in vitro* polarized Th17-cells is a conventional approach to studying the Th17 immune response *in vivo* and assumes that the inflammatory response following antigen challenge will recapitulate the antigen-specific, endogenously-generated response. This Th17 adoptive transfer model was used to demonstrate the critical nature of Th17 in glucocorticoid-resistant allergic airway disease [32] We therefore sought to compare the *in vivo* generated immune response in NO_2_-promoted allergic airway disease to that generated following the adoptive transfer of OVA-specific *in vitro* polarized Th17 cells. We first observed that the magnitude of the inflammatory response induced following adoptive transfer much greater than that elicited by the endogenous Th17 response in NO_2_-promoted allergic airway disease. It has been reported that the adoptive transfer of Th17 *in vitro* polarized D011.10 cells are capable of inducing the production of IL-13 following antigen challenge *in vivo* [31]. The cytokine induction upon restimulation of lung cells in the presence of OVA was between 3-fold to 10-fold for Th17 cytokines following Th17 adoptive transfer and 6-fold to 30-fold higher for Th2 cytokines following Th2 adoptive transfer, indicating that the potential cytokine production *in vivo* following adoptive transfer is substantially higher in comparison to the endogenously-generated Th17 and Th2 responses. This can easily be explained by the fact that OTII cells all express an OVA-specific TCR, and thus every cell is potentially activated, whereas the number of OVA-specific CD4^+^ T cells generated endogenously is much smaller. However, this observation does not address the discrepancies we uncovered in the sensitivity to Dex and anakinra between Th17 adoptive transfer and NO_2_-promoted allergic airway disease. For example, we observed no evidence that increasing the *in vitro* dose of anakinra resulted in further diminution of IL-17A or further suppression by Dex (Figure S2A). Considering that anakinra is a competitive antagonist of IL-1R ligand binding [88], the resistance observed following Th17 adoptive transfer cannot be explained by magnitude alone.

In agreement with previous reports [18,32], we observed that IL-17 production *ex vivo* by lung cells following Th17 adoptive transfer and antigen challenge was resistant to inhibition by Dex, whereas IL-17 production by lung cells from mice sensitized by NO_2_ was Dex-sensitive. This distinction demonstrates substantial differences in the behavior of the Th17 response between these models. In a model of allergic airway disease in which mice were sensitized via a mucosal route, the endogenously-generated Th17 response exhibited Dex sensitivity under conditions of chronic antigen challenge, in contrast to Dex-resistant *in vitro* polarized OTII Th17 cells [60]. The authors attribute the *in vivo* Dex sensitivity to inhibition of Th17 cell expansion, whereas established Th17 effector cells may be Dex resistant [60]. Because our system tested lung cells following OVA challenge, we expect that resident lymphocytes at the time point analyzed included antigen-specific effector T-cells that had been recruited to the lung as a consequence of antigen challenge. Thus, the observed differences in Dex sensitivity are likely not due to T-cell maturation status.

The adoptive transfer of Th17 *in vitro* polarized T-cells or allergic airway disease elicited in *Rorγt*-overexpressing mice consists of a Th17-predominate response that is also resistant to treatment with Dex [18,32]. Because cytokines of the Th2 immune response are capable of influencing sensitivity to Dex [59], we also sought to control for the possibility that the presence of a Th2 response might impact Dex sensitivity. The Dex sensitivity of the endogenously-generated Th17 response did not change following inhibition of the generation of a Th2 response. Furthermore, IL-17A production by lung cells from mice that received the adoptive transfer of *in vitro* polarized Th2 cells and were then antigen challenged was also sensitive to Dex treatment, demonstrating that *in vitro* culture prior to adoptive transfer was not responsible for Dex-resistant IL-17 production. It is interesting that the Dex and anakinra sensitivity profiles of restimulated lung cells following NO_2_-promoted allergic sensitization and challenge matched closely with those of the Th2 adoptive transfer. Considering that the STAT6-/- mouse exhibited no pulmonary inflammation in our model, it may be that the Th17 cytokine-producing cells in NO_2_-promoted allergic airway disease are in fact Th2 cells [81]. A recent article described that *in vitro* sensitivity to Dex can recapitulate sensitivity *in vivo*, validating the functional relevance of our approach [89]. Thus, the finding that the endogenously-generated Th17 response is sensitive to Dex *ex vivo* refutes the hypothesis that IL-17 contributes to Dex resistance *in vivo*.

IL-23 and IL-17 have been demonstrated to increase the expression of GC receptor beta, a dominant negative isoform of the GC receptor, in PBMCs from healthy human donors [90], thereby providing a mechanism through which these cytokines can induce Dex resistance. On the other hand, IL-17 does not necessarily correlate with Dex resistance in human samples [91]. In mouse models of allergic airway disease, the few studies reporting on Dex responsiveness of the endogenously-generated Th17 response indicate that that it is Dex sensitive [92-95]. RSV infection at the time of antigen challenge results in increased levels of IL-17A and AHR development that is exacerbated by Dex treatment, suggesting that in the context of exacerbation, IL-17A may contribute to Dex resistance [25,96].

Similar to the Dex studies, blocking the Th2 response with anti-IL-4 did not change the sensitivity of the endogenously-generated Th17 response to treatment with anakinra. The slight, albeit statistically significant, diminution in some Th17 cytokines by anakinra might have reflected the fact that while the generation of *in vitro* polarized Th17 cells is independent of IL-1R signaling, IL-1R signaling contributes to the expansion of these cells [19]. Nonetheless, adoptive transfer of *in vitro* polarized Th17 cells resulted in an anakinra-resistant phenotype, indicating that the immune response generated *in vivo* following Th17 adoptive transfer is likely independent of IL-1R signaling.

Taken together, our data support a nonpathogenic role for IL-17A and a protective role for the IL-1R-dependent Th17 adaptive immune response generated as a consequence of NO_2_-promoted allergic airway disease. This response likely does not contribute to glucocorticoid resistance *in vivo*. Furthermore, the endogenously-generated Th17 response is qualitatively distinct from that generated as a result of adoptive transfer of *in vitro* polarized Th17 cells, both in its sensitivity to GC and its requirement for IL-1R signaling. These data demonstrate that the Th17 response generated endogenously is substantially different than the response acquired upon adaptive transfer of *in vitro* polarized Th17 cells and OVA challenge, suggesting that in some instances, the outcomes of an adoptive transfer study may not be generalizable to other models of allergic airway disease. Clarification of where these models fit in terms of clinical asthma demands robust comparative analyses [97]. As exemplified by our findings, the functional significance of the Th17 response in promoting allergic airway disease is determined by the conditions in which it was generated, ranging from protective to pathogenic. Given the heterogeneity among patients with clinical asthma, the contribution of a single type of immune response may also vary among patients, which should be considered during pharmacologic development and clinical testing [60].

## Supporting Information

Figure S1 **NO_2_-dependent IgG exacerbation of AHR is independent of the inflammatory response.** Mice received IgG isotype control antibody (1 mg in saline) one day prior to NO_2_ exposure on day 0 and again one day prior to antigen challenge on day 13, while NO_2_/O mice received no antibody. Mice were exposed to NO_2_ on day 1 (NO_2_/O and IgG/NO _2_/O groups only). IgG/O mice received IgG antibody and were exposed to OVA, but not NO_2_. Non-inflamed negative control mice (non-inf) were naïve to antigen. All experimental groups were exposed to OVA on days 1-3 and again during the antigen challenge on days 14-16. 48 hours following the final antigen challenge, methacholine responsiveness was determined. Percent baseline and average percent baseline per dose of methacholine were calculated for R (A-B) and E (C-D). BAL total cells (E), macrophages (Macs; F), neutrophils (PMNs; G), and eosinophils (Eos; H) were determined. Lungs were removed, enzymatically digested, and restimulated for 96 hours in the presence of IgG alone (10 μg/mL), OVA, or IgG and OVA for 96 hours prior to cytokine analysis by ELISA (I-K). Statistics were performed by 2-way ANOVA (B and D) or 1-way ANOVA (E-H) and Bonferroni post hoc analysis. **** p < 0.0001, *** p < 0.001, ** p < 0.01, * p < 0.05 compared to non-inflamed unless otherwise indicated by brackets. ND, not significantly different compared to non-inflamed, unless otherwise indicated. n=6 (A-F) or n=3 (G-I) per group. The dashed line in J and K represents the lower limit of quantitation in the assays.
(TIFF)

Figure S2 **Increasing the dose of anakinra does not significantly impact cytokine production by lung cells from Th17 adoptively transferred and OVA challenged recipient mice.** CD4^+^ T-cells from OTII mice were either Th2 or Th17 polarized *in* *vitro* and adoptively transferred to recipient mice, which were then OVA-challenged for 3 consecutive days and analyzed 24 hours following the final OVA challenge. At analysis, lungs were removed and enzymatically digested. Lung single-cell suspensions were restimulated with OVA antigen and incubated with increasing concentrations of anakinra and Dex, as indicated. Cell supernatants were harvested at 96 hours and analyzed for the production of IL-17A (A) or IL-5 (B) by ELISA. Statistics were performed by 2-way ANOVA and Bonferroni post hoc analysis for cytokine production resulting from increasing doses of anakinra. * p < 0.01 compared to the 0 μg/mL anakinra dose for that particular dex dose; **^◊^** p < 0.05 compared to 0 M Dex for the indicated anakinra dose; **^†^** p < 0.01 compared to 10^-9^ M Dex for the indicated anakinra dose; ^‡^ p < 0.0001 compared to 10^-8^ M for the indicated anakinra dose. For the Dex effect per dose of anakinra, statistically significant decreases in cytokine production are shown only for the corresponding 10-fold increase in Dex concentration. The dashed line in A represents the upper limit of detection of the assay. Samples from n = 4 mice were pooled prior to *in* *vitro* restimulation, which was performed in triplicate.
(TIFF)

File S1 **Supporting Materials & Methods, Results and References.**
(DOCX)
