# Supplementary figures and images for: The Endogenous Th17 Response in NO2-Promoted Allergic Airway Disease Is Dispensable for Airway Hyperresponsiveness and Distinct from Th17 Adoptive Transfer
Source: PLoS One. 2013 Sep 19;8(9):e74730. doi: 10.1371/journal.pone.0074730 (PMC3778003; doi:10.1371/journal.pone.0074730)

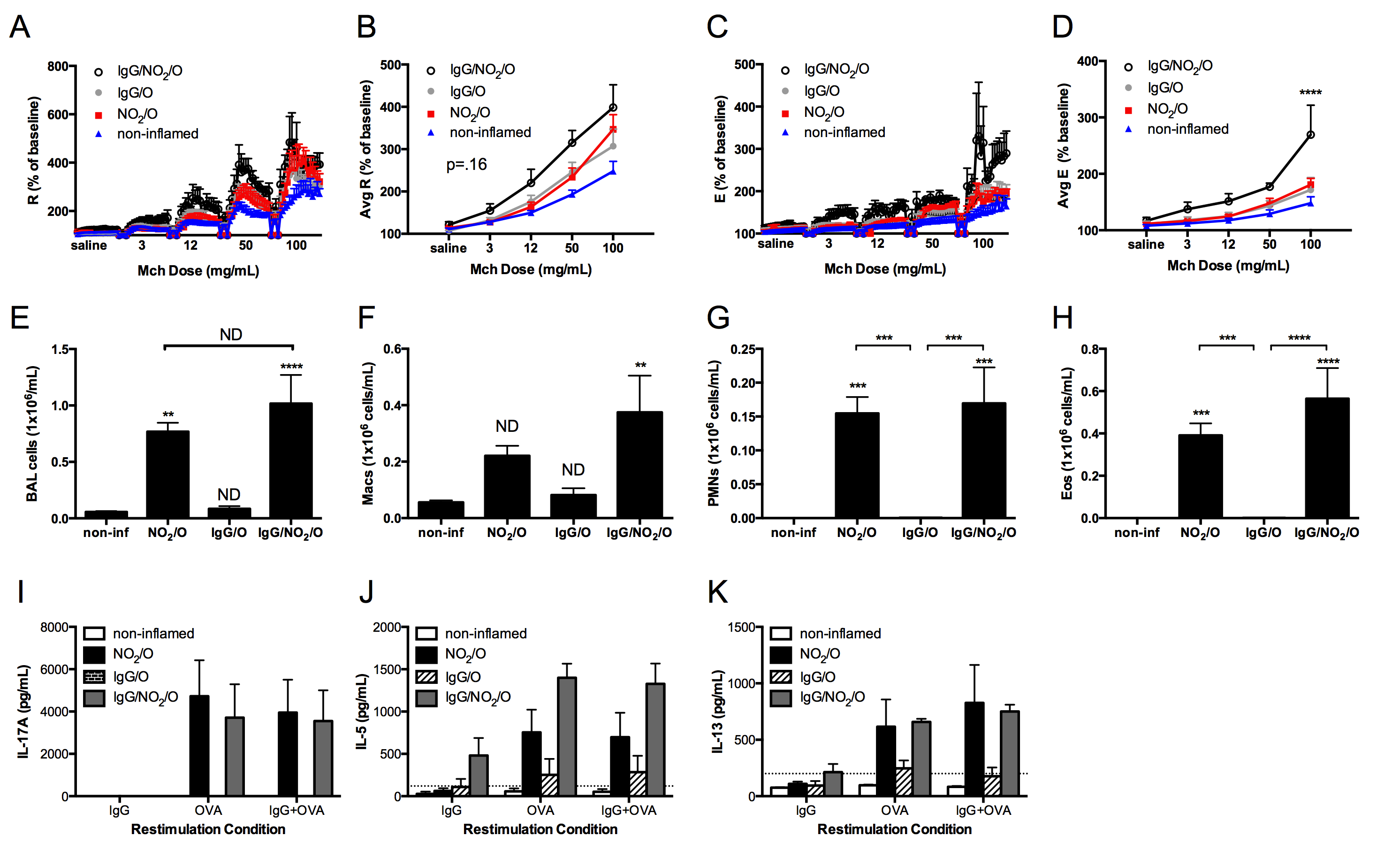

Supplement: Figure S1 — NO2-dependent IgG exacerbation of AHR is independent of the inflammatory response. Mice received IgG isotype control antibody (1 mg in saline) one day prior to NO2 exposure on day 0 and again one day prior to antigen challenge on day 13, while NO2/O mice received no antibody. Mice were exposed to NO2 on day 1 (NO2/O and IgG/NO 2/O groups only). IgG/O mice received IgG antibody and were exposed to OVA, but not NO2. Non-inflamed negative control mice (non-inf) were naïve to antigen. All experimental groups were exposed to OVA on days 1-3 and again during the antigen challenge on days 14-16. 48 hours following the final antigen challenge, methacholine responsiveness was determined. Percent baseline and average percent baseline per dose of methacholine were calculated for R (A-B) and E (C-D). BAL total cells (E), macrophages (Macs; F), neutrophils (PMNs; G), and eosinophils (Eos; H) were determined. Lungs were removed, enzymatically digested, and restimulated for 96 hours in the presence of IgG alone (10 μg/mL), OVA, or IgG and OVA for 96 hours prior to cytokine analysis by ELISA (I-K). Statistics were performed by 2-way ANOVA (B and D) or 1-way ANOVA (E-H) and Bonferroni post hoc analysis. **** p < 0.0001, *** p < 0.001, ** p < 0.01, * p < 0.05 compared to non-inflamed unless otherwise indicated by brackets. ND, not significantly different compared to non-inflamed, unless otherwise indicated. n=6 (A-F) or n=3 (G-I) per group. The dashed line in J and K represents the lower limit of quantitation in the assays. (TIFF) [file pone.0074730.s001.tiff]

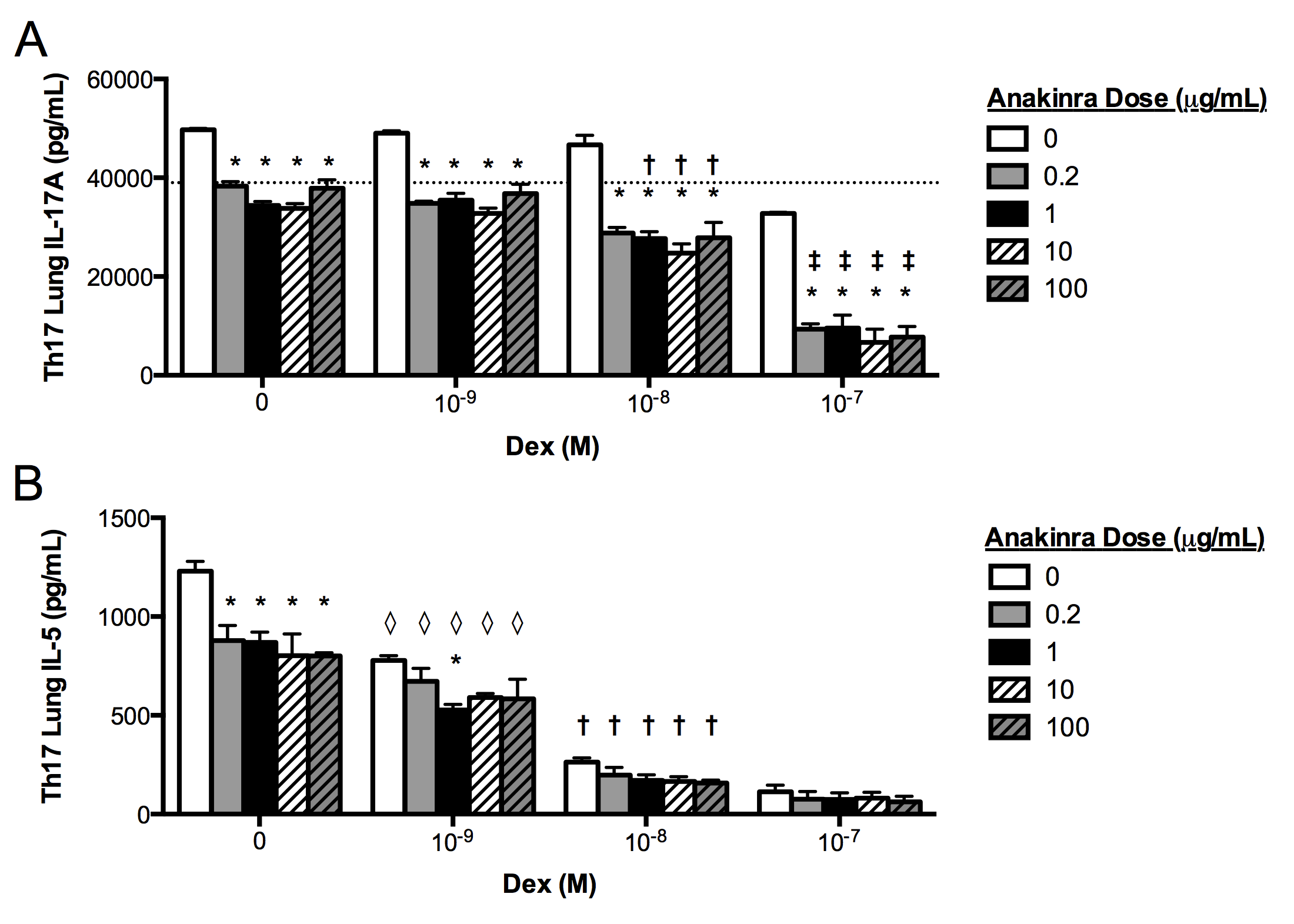

Supplement: Figure S2 — Increasing the dose of anakinra does not significantly impact cytokine production by lung cells from Th17 adoptively transferred and OVA challenged recipient mice. CD4+ T-cells from OTII mice were either Th2 or Th17 polarized in vitro and adoptively transferred to recipient mice, which were then OVA-challenged for 3 consecutive days and analyzed 24 hours following the final OVA challenge. At analysis, lungs were removed and enzymatically digested. Lung single-cell suspensions were restimulated with OVA antigen and incubated with increasing concentrations of anakinra and Dex, as indicated. Cell supernatants were harvested at 96 hours and analyzed for the production of IL-17A (A) or IL-5 (B) by ELISA. Statistics were performed by 2-way ANOVA and Bonferroni post hoc analysis for cytokine production resulting from increasing doses of anakinra. * p < 0.01 compared to the 0 μg/mL anakinra dose for that particular dex dose; ◊ p < 0.05 compared to 0 M Dex for the indicated anakinra dose; † p < 0.01 compared to 10-9 M Dex for the indicated anakinra dose; ‡ p < 0.0001 compared to 10-8 M for the indicated anakinra dose. For the Dex effect per dose of anakinra, statistically significant decreases in cytokine production are shown only for the corresponding 10-fold increase in Dex concentration. The dashed line in A represents the upper limit of detection of the assay. Samples from n = 4 mice were pooled prior to in vitro restimulation, which was performed in triplicate. (TIFF) [file pone.0074730.s002.tiff]
