# Supplementary material for: The Endogenous Th17 Response in NO2-Promoted Allergic Airway Disease Is Dispensable for Airway Hyperresponsiveness and Distinct from Th17 Adoptive Transfer
Source: PLoS One. 2013 Sep 19;8(9):e74730. doi: 10.1371/journal.pone.0074730 (PMC3778003; doi:10.1371/journal.pone.0074730)
Supplement: File S1 — Supporting Materials & Methods, Results and References. (DOCX) [file pone.0074730.s003.docx]

**The Endogenous Th17 Response in NO_2_-Promoted Allergic Airway Disease Is Dispensable for Airway Hyperresponsiveness and Distinct from Th17 Adoptive Transfer**

Rebecca A. Martin, Jennifer L. Ather, Rebecca Daggett, Laura Hoyt, John F. Alcorn, Benjamin T. Suratt, Daniel J. Weiss, Lennart K.A. Lundblad, and Matthew E. Poynter

## Supporting Information

### Materials and methods S1

#### Mice

For the acute IgG studies, 13-26 week old male and female mice on the C57BL/6 background and bred at the University of Vermont, some of which contained the reverse tetracycline transactivator protein expressed in non-ciliated airway epithelial cells (CC10-rtTA), which is not active in the absence of doxycycline [[1](#_ENREF_1)], or for the immunohistochemistry and neutrophil depletion studies, female C57BL/6 mice age 6-8 weeks from Jackson Laboratories (Bar Harbor, ME), were used. Studies were approved by the Institutional Animal Care and Use Committee of the University of Vermont under the permits 09-050, 12-018, and 12-020. All mice were euthanized with sodium pentobarbitol (200-300 μL by i.p. injection; Wilcox Pharmacy, Rutland, VT) or for pulmonary function assessment, anesthetized with 90 mg/kg sodium pentobarbital.

#### In vitro IgG treatments

To assess whether IgG promotes the production of acute inflammatory mediators *in vitro*, splenocytes from non-inflamed C57BL/6 mice were were generated by passing the spleens through a 70 μm nylon mesh filter (BD Biosciences) and mononuclear leukocytes were enriched by separation with Lymphocyte Separation Medium (MP Biomedicals, Irvine, CA). Single cell suspensions were plated at 1x10^6^ cells/mL in CD4^+^ complete media for 24 hours with 1, 10, or 50 μg/mL IgG1 (MOPC21, BioXCell) or IgG2a (2A3, BioXCell). Positive control splenocytes were stimulated with 1 μg/mL LPS (Invivogen, San Diego, CA). Negative control cells were incubated in media alone. For the study assessing IgG in NO_2_-promoted allergic airway disease, to *in vitro* antigen restimulation of lung cells, 10 μg/mL IgG (MOPC21, BioXCell) was added in the presence or absence of 400 μg/mL OVA (Sigma, St. Louis, MO).

#### In vivo antibody treatments

For acute studies, mice received either 1 mg IgG (MOPC21, BioXCell), 1 mg IgG2a (2A3, BioXCell), or saline, followed by 30 minutes of nebulized 1% OVA, Fraction V (Sigma-Aldrich, St. Louis, MO) in saline and analyzed at 24 hours. For *in vivo* treatment of mice in the immunohistochemical analysis, mice received 1 mg IgG (MOPC21, BioXCell) in saline. 24 hours after IgG administration, mice were exposed to air or 15ppm of NO_2_ for 1 hour followed by 30 minutes of nebulized 1% OVA, Fraction V (Sigma-Aldrich) in saline. Analyses were conducted 24 hours following the OVA exposure. To assess the effect of IgG in NO_2_-promoted allergic airway disease, mice received 1 mg IgG (MOPC21, BioXCell) on day 0, one day prior to NO_2_-promoted sensitization, and on day 13, one day prior to antigen challenge. Another group of mice was not exposed to NO_2_, but received all IgG administrations and was exposed to OVA antigen. Positive control mice did not receive IgG, and were NO_2_-allergically sensitized and antigen challenged. Negative control mice were untreated and non-inflamed. For *in vivo* neutrophil depletion studies, 0.5 mg anti-Ly6G (1A8, BioXCell) or IgG2a (2A3, BioXCell) isotype control antibody was administered on day 13. Antigen challenge was performed for 3 consecutive days on day 14, 15, and 16, and analysis was conducted 48 hours following the final antigen challenge on day 18.

*Serum collection*

Following euthanasia, blood was collected via cardiac puncture of the right ventricle and placed into a serum separator tubes (BD Biosciences, San Diego, CA). Following centrifugation at 13,200 rpm for 10 minutes, serum was collected for cytokine analysis.

*Cytokine quantitation for acute IgG studies*

All cytokine analyses for the acute IgG studies were conducted with a Milliplex assay (Millipore), according to manufacturer’s instructions.

*Immunohistochemical analysis*

At analysis, lungs were removed, inflated with OCT (Triangle Biomedical Sciences, Durham, NC), and snap frozen. Lung sections were briefly rinsed in PBS to remove media, fixed in fresh 3% paraformaldehyde (Sigma) and stained with goat anti-mouse IgG1 conjugated to alexa fluor 647 (Invitrogen, Grand Island, NY) in 1% bovine serum albumin (Fisher Scientific, Waltham, MA). Alternatively, slides were fixed with zinc formalin (z-fix; Anatech, Ltd., Battle Creek, MI) and assessed using alexa fluor 647 tyramide amplification (Molecular Probes, Eugene, OR) after staining with goat anti-mouse IgG1 conjugated to alexa fluor 647 (Invitrogen, Grand Island, NY) and donkey anti-goat horseradish peroxidase (Jackson Immunoresearch, West Grove, PA). All sections were counterstained with DAPI (Invitrogen) and mounted with Vector mounting media (Vector Laboratories, Burlington, CA). Cryostat sectioning and imaging on a BX50 light microscope (Olympus, Center Valley, PA) were performed with the assistance of UVM’s Microscopy Imaging Center.

### Results S1

To test the possibility that IgG administration at sensitization and challenge augments AHR, we treated mice with IgG control antibody one day prior to NO_2_-promoted sensitization on day 0 and one day prior to antigen challenge on day 13. To determine whether IgG administration in the absence of NO_2_ was sufficient to augment methacholine (MCh) responsiveness, one group of mice received IgG treatments and was exposed to OVA, but not NO_2_. Positive control mice were subjected to NO_2_-promoted allergic sensitization and antigen challenged, but received no antibody. We observed that IgG administered at the time of sensitization and challenge resulted in a statistically significant increase in AHR at 50 mg/mL MCh compared with NO_2_-sensitized and challenged mice that did not receive IgG isotype control antibody (Fig. S1A-D). We found that IgG administration at the time of antigen challenge alone did not impact AHR development (Fig. 1E-H). Interestingly, this effect of IgG isotype control antibody administration was only evident in the context of NO_2_ sensitization, as we did not observe increased AHR in mice that received IgG antibody and were exposed to OVA but not NO_2_. In NO_2_-sensitized and challenged mice, BAL neutrophils and eosinophils were elevated above that of non-inflamed mice and mice that received IgG and OVA exposures in the absence of NO_2_ (Fig. S1E-H). BAL cellularity did not differ between IgG treated NO_2_-sensitized and challenged mice and mice that were NO_2_-sensitized and challenged but did not receive IgG antibody. Furthermore, production of IL-17A, IL-5, and IL-13 by lung cells from NO_2_-sensitized and challenged mice following restimulation in the presence of OVA was similar to that from IgG-treated NO_2_ sensitized and challenged mice (Fig. S1I-K). In contrast, antigen-restimulated lung cells from IgG treated and OVA exposed mice that were not exposed to NO_2_ produced similar levels of cytokines to non-inflamed negative control mice. Incubation of lung cells with IgG did not result in increased cytokine production, and incubation of antigen-restimulated lung cells with IgG did not augment cytokine production (Fig. S1I-K). These data suggest a combined effect of IgG treatment and NO_2_ sensitization on AHR, but not on airway inflammation. Furthermore, cytokine analysis indicates that augmentation of AHR is not a result of an antigen-specific response against IgG.

On further analysis, we did not detect any acute mediators of inflammation in the serum or BAL of mice 24 hours following IgG administration or in cell supernatants following the the *in vitro* exposure of splenocytes to IgG (data not shown), suggesting that this isotype control IgG antibody does not elicit adjuvant effects or activate an innate immune response. In serum, MIG (monokine induced by IFNγ or CXCL9) was significantly increased over vehicle injected mice, but was not statistically increased over a group of mice that received a rat IgG2a isotype control antibody. Furthermore, IP-10, Eotaxin, KC, Rantes, IL-5, MIP-1α, MIP-1β, MIP-2, IL-4, IL-7, and IL-10 were not different from vehicle-injected mice. MIP-1β, IL-13, and IFNγ were not detected (Milliplex; n = 5-8/group). In BAL, IgG-treated mice were not significantly different from vehicle treated mice for IP-10, IL-10, KC, and MIG in BAL samples, and eotaxin, MCP-1, MIP-1α, MIP-1β, MIP-2, RANTES, IL-17, IL-13, IL-5, IL-4, IFNγ, eotaxin were not detected in any samples. In our *in vitro* assays (n=3/group), LPS-stimulated splenocytes produced IL-10, MIP-1α, KC, IFNγ, MIP-1β, RANTES, IP-10, and MIP-2. However, IgG-stimulated splenocytes did not produce these cytokines. Additional cytokines that were not detected in any samples included IL-4, IL-5, IL-13, IL-17, MCP-1, eotaxin, and MIG.

In addition, we did not detect increased IgG deposition by immunohistochemical analysis of lungs from mice that were injected with IgG and subsequently exposed to NO_2_ in comparison with lungs from mice that were injected with IgG but not exposed to NO_2_ (data not shown), suggesting that the augmentation of AHR is not due to IgG’s detection of unveiled or newly-exposed antigenic epitopes following NO_2_ exposure. We therefore conclude that the isotype control-mediated increase in AHR is secondary to an undefined mechanism that is likely independent of the immune response.

**Reference S1**

1. Ather JL, Alcorn JF, Brown AL, Guala AS, Suratt BT, et al. (2010) Distinct functions of airway epithelial nuclear factor-kappaB activity regulate nitrogen dioxide-induced acute lung injury. Am J Respir Cell Mol Biol 43: 443-451.
